# Real-Time High-Sensitivity Reaction Monitoring of Important Nitrogen-Cycle Synthons by ^15^N Hyperpolarized Nuclear Magnetic Resonance

**DOI:** 10.1021/jacs.2c02619

**Published:** 2022-05-04

**Authors:** Peter J. Rayner, Marianna Fekete, Callum A. Gater, Fadi Ahwal, Norman Turner, Aneurin J. Kennerley, Simon B. Duckett

**Affiliations:** † Centre for Hyperpolarisation in Magnetic Resonance, Department of Chemistry, University of York, Heslington, York YO10 5DD, U.K.; ‡ Department of Engineering and Technology, University of Huddersfield, Queensgate, Huddersfield, West Yorkshire HD1 3DH, U.K.

## Abstract

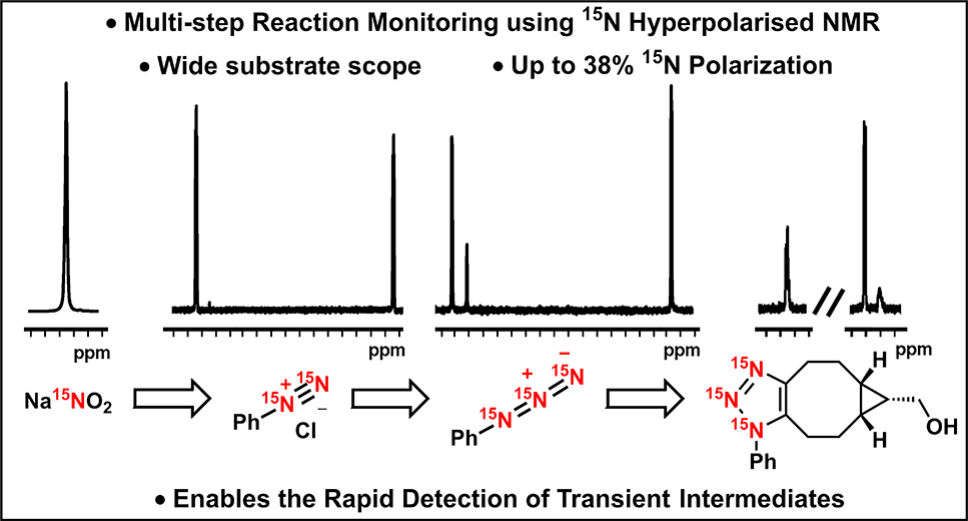

Here, we show how signal amplification by reversible exchange hyperpolarization of a range of ^15^N-containing synthons can be used to enable studies of their reactivity by ^15^N nuclear magnetic resonance (NO_2_^–^ (28% polarization), ND_3_ (3%), PhCH_2_NH_2_ (5%), NaN_3_ (3%), and NO_3_^–^ (0.1%)). A range of iridium-based spin-polarization transfer catalysts are used, which for NO_2_^–^ work optimally as an amino-derived carbene-containing complex with a DMAP-*d*_2_ coligand. We harness long ^15^N spin-order lifetimes to probe in situ reactivity out to 3 × *T*_1_. In the case of NO_2_^–^ (*T*_1_ 17.7 s at 9.4 T), we monitor PhNH_2_ diazotization in acidic solution. The resulting diazonium salt (^15^N-*T*_1_ 38 s) forms within 30 s, and its subsequent reaction with NaN_3_ leads to the detection of hyperpolarized PhN_3_ (*T*_1_ 192 s) in a second step via the formation of an identified cyclic pentazole intermediate. The role of PhN_3_ and NaN_3_ in copper-free click chemistry is exemplified for hyperpolarized triazole (*T*_1_ < 10 s) formation when they react with a strained alkyne. We also demonstrate simple routes to hyperpolarized N_2_ in addition to showing how utilization of ^15^N-polarized PhCH_2_NH_2_ enables the probing of amidation, sulfonamidation, and imine formation. Hyperpolarized ND_3_ is used to probe imine and ND_4_^+^ (*T*_1_ 33.6 s) formation. Furthermore, for NO_2_^–^, we also demonstrate how the ^15^N-magnetic resonance imaging monitoring of biphasic catalysis confirms the successful preparation of an aqueous bolus of hyperpolarized ^15^NO_2_^–^ in seconds with 8% polarization. Hence, we create a versatile tool to probe organic transformations that has significant relevance for the synthesis of future hyperpolarized pharmaceuticals.

## Introduction

Positron emission tomography (PET) is a very sensitive technique that uses gamma cameras to image changes in metabolic processes, blood flow, and agent absorption in the body. It takes long-lived radionuclides generated using a cyclotron that are then embedded into a suitable receptor to create the radiopharmaceuticals that convey the diagnostic response. Unfortunately, this process can be complex and costly. Magnetic resonance imaging (MRI) is another powerful diagnostic method, but inherent low sensitivity means that routine clinical measurements probe highly abundant water.

Consequently, there has been a great deal of excitement in the clinical community with a method called hyperpolarization that gives MRI the sensitivity needed to visualize changes in metabolic flux by detecting biomolecules that encode disease. The most clinically developed method currently involves the use of dissolution dynamic nuclear polarization (*d*-DNP).^1−3^ One alternative method to create hyperpolarization is *para*hydrogen (*p*-H_2_)-induced polarization (PHIP), which despite being discovered in the 1980s is only now receiving worldwide attention. Three recent significant PHIP advances that utilize *p*-H_2_ are signal amplification by reversible exchange (SABRE),^4^*p*-H_2_-induced polarization with side-arm hydrolysis,^5^ and the rapid hyperpolarization and purification of the metabolite fumarate.^6,7^ As *p*-H_2_ can be prepared to a level of 50% purity by simply cooling H_2_ gas by liquid nitrogen,^8^ one could imagine the widespread future use of this MR sensitization approach.

Here, we demonstrate how it is possible to turn PHIP into a versatile tool for the in situ synthesis of a family of long-lived and highly MR visible precursors containing ^15^N, akin to the radionuclides of PET. These reactive intermediates are rapidly embedded into important molecular reporters to illustrate the creation of the hyperpharmaceutical. We achieve this by harnessing reactive species like nitrite (NO_2_^–^), nitrosonium (NO^+^), and ammonia (NH_3_), representing simple building blocks which can be transformed into a wide range of hyperpolarized materials.

Our method takes their ^15^N isotopologues and uses PHIP to first hyperpolarize them. Thus we create an imbalance in one of the ^15^N’s two possible nuclear spin orientations (+1/2 or −1/2) which can potentially be maintained for 10’s of minutes if stored in an appropriate magnetic field.^9−11^ We focus on establishing this concept by reference to nuclear magnetic resonance (NMR), a technique that is used by many scientific disciplines. NMR mainly detects ^1^H responses, because the most commonly probed alternative nucleus, ^13^C, is 6400 times harder to detect than ^1^H. This is due to ^13^C’s 1% abundance and small gyromagnetic ratio (γ). Consequently, the Zeeman splitting yielding the resonance frequency is four times smaller than ^1^H, and a minute macroscopic nuclear magnetization occurs, which is detected by NMR.

Less utilized ^15^N has a highly informative 1350 ppm chemical shift range and long *T*_1_,^9^ but as ^15^N is only 0.36% abundant and has a γ 10 times smaller than ^1^H, it is 260,000 times harder to detect. Hence, high-concentration samples and extensive signal averaging are needed for NMR studies at natural abundance. Despite this limitation, nitrite ions have been probed by ^15^N NMR in solution and the solid state^12^ and used to study chemodenitrification in humic substances^13^ and nitric oxide release from copper sites, so its utility is established.^14^ Importantly, the ^15^N isotope can be sourced cheaply in materials like ^15^NH_4_Cl and Na^15^NO_2_, and this offers routes to synthesize other isotopically labeled compounds such as pharmaceuticals. Hyperpolarized Na^15^NO_2_, created by *d*-DNP, has been studied.^15^

The sensitivity gains provided by PHIP have already been used widely to aid the study of organic and inorganic chemicals, and it has made the detection of previously hidden intermediates possible.^16−19^ Our aim here is to illustrate the hyperpharmaceutical concept while establishing that we can track chemical reactivity, complete the diagnostic fingerprinting of materials, and dramatically expand chemical diversity in the field of hyperpolarization.

We start with nitrite (NO_2_^–^), a reagent that is formed during the nitrification of ammonia by nitrosomas within the nitrogen cycle.^20^ While mammals do not absorb nitrites directly, plants use it to form essential nitrogen-containing molecules such as amino acids and further aerobic oxidation of nitrite leads to nitrate. Nitrite is used as a food additive for cured meats^21^ and approximately 7% of our ingested nitrite comes this source, while the remainder comes from the enterosalivary pathway.^3,22^ While nitrites are noncarcinogenic, their ability to form nitrosamines can lead to toxicity^23^ as examined by the research community and mainstream media.^24,25^ The action of methmyogolbin production by nitrite is, however, beneficial in the treatment of cyanide poisoning and sodium nitrite remains as one of the primary antidotes for acute intoxication.^26^

The nitrite ion, usually as sodium nitrite, finds widespread use in the chemical industry, because of its oxidizing properties and role in organic transformations; common examples are the Sandmeyer reaction, which transforms aryl amines into aryl halides, and the diazotization reaction that is used en route to the formation of dyes and pigments.^27^

Nitrite is also an ambidentate ligand that can bind to metals via the N- or O-atoms to form nitro or nitrito complexes, respectively,^28,29^ with Ni^30−33^ and Pt^34−36^ examples being the most prevalent. As the PHIP hyperpolarization method SABRE works through reversible binding of the agent that is set to become hyperpolarized to a metal complex, we hypothesized that polarization of NO_2_^–^ via such a route is possible.^4,11,37,38^

In fact, there are a few examples of ionic species such as sodium pyruvate,^39,40^ sodium acetate,^41^ and naicin^42^ that undergo SABRE. This method requires the creation of a scalar coupling network between the target agent and *p*-H_2_-derived protons in a catalyst.^43−46^ Hence, an η^1^-NO_2_ (*N*-nitro) complex with a potentially large hydride-^15^N coupling would be preferred over η^1^-ONO (*O*-nitrito) or η^2^-O–N–O (*O*,*O*-bidentate) linkage isomers. Theoretical descriptions of SABRE are provided by Barskiy and others^43,44,47^ and account for the magnetization transfer conditions needed to sensitize a range of agents.^45^ Transfer is optimized at low magnetic fields, typically 6 mT for ^1^H, or through *r.f*. excitation at high field.^48^ When combined with suitable catalyst lifetimes, this has driven the efficient sensitization of ^1^H, ^13^C, ^15^N, ^19^F, ^31^P, and ^29^Si (etc.)^39−41,49−59^ nuclei.

Here, we also evaluate the azide anion, an excellent nucleophile that readily forms organic azides such as the antiretroviral AZT, Avapro, Diova, and Tamiflu. This functionality can be readily reduced to create amines, and through the Curtius rearrangement carbamates. Copper-catalyzed azide-alkyne cycloadditions or click reactions are also important. Consequently, azide represents an important precursor to agrochemicals, pharmaceuticals, and natural products so its successful hyperpolarization is also desirable.

Typically, when an iridium *N*-heterocyclic carbene (NHC) catalyst is used, products can be created whose NMR signal strengths are many orders of magnitude higher than those which would be obtained at thermal equilibrium.^60,61^ Warren et al. in particular stand-out for their work on ^15^N^68^ in a refinement called SABRE-SHEATH,^51,56^ and up to 79% ^15^N polarization has recently been reported for a range of neutral Lewis bases.^62^ Several alternative radio-frequency transfer strategies have also been exemplified,^48,63,64^ and given one of the goals of SABRE is in vivo detection, water-soluble SABRE catalysts have been described,^65,66^ with the in vitro MRI detection of an ^15^N response already illustrated.^66^ Tessari et al. have developed a number of analytical science applications for SABRE^67^ and other catalyst types have been reported.^68^ Furthermore, hyperpolarized long-lived singlet states, as pioneered by Levitt,^69^ have been created and detected after their formation.^50,70−74^ Consequently, we might expect the benefits of such a simple approach to sensitize a range of ^15^N-containing reagents to be substantial.

## Results and Discussion

### Demonstration That an Active SABRE Catalyst Forms with Na^15^NO_2_

As indicated, for successful SABRE transfer to occur, the formation of a complex exhibiting spin–spin couplings between the bound substrate and *p*-H_2_-derived hydride nuclei is required. Classically, this involves the reaction of a precatalyst (most commonly [IrCl(COD)(IMes)] (**1**) (IMes = 1,3-bis(2,4,6-trimethylphenyl)imidazolylidene)), with an excess of the selected substrate under a H_2_ atmosphere. Complexes of type [Ir(H)_2_(IMes)(sub)_3_]Cl, when the substrate is a neutral *N*-heterocycle such as pyridine, meet this requirement.^4^ Consequently, our initial efforts targeted the synthesis of an active SABRE catalyst with bound NO_2_^–^ rather than pyridine.

**Figure 1:**
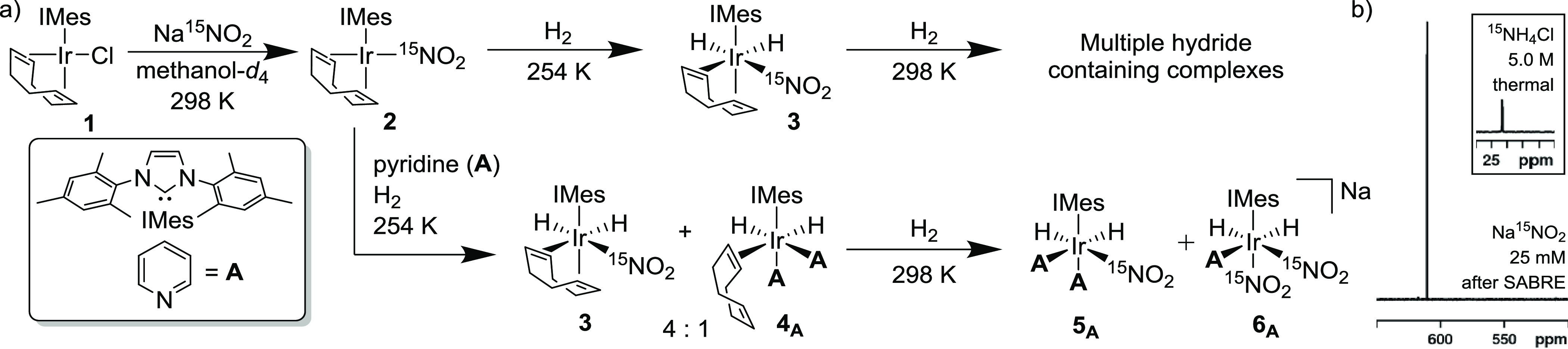
(a) Reaction of [IrCl(COD)(IMes)] with Na^15^NO_2_ in the presence of hydrogen and pyridine (inset: structure of the IMes and pyridine ligand). (b) ^15^N NMR spectrum of Na^15^NO_2_ after SABRE hyperpolarization under 3 bar *p*-H_2_ (inset: thermally polarized ^15^N NMR spectrum of a 5.0 M solution of ^15^NH_4_Cl in D_2_O for comparison).

When Na^15^NO_2_ (1 equiv) was added to a solution of [IrCl(COD)(IMes)] (**1**, 5 mM) in methanol-*d*_4_, the complete conversion to [Ir(^15^NO_2_)(COD)(IMes)] (**2**) at 298 K (Figure 1a) is observed. This change was readily evident as the ^15^N signal for free Na^15^NO_2_ at δ_N_ 611.8 moved to δ_N_ 490.7 for the bound NO_2_^–^ at 255 K (see the Supporting Information). When **2** was then exposed to a 3 bar pressure of H_2_ at 254 K, the oxidative addition of hydrogen took place to form [Ir(H)_2_(^15^NO_2_)(COD)(IMes)] (**3**). This complex exhibits ^1^H NMR for its hydride ligands at δ_H_ −18.77 (hydride *trans* to ^15^NO_2_^–^, ^2^*J*_HN_ = 23.1 Hz and ^2^*J*_HH_ = 3.3 Hz) and δ_H_ −14.17 (hydride *trans* to COD, ^2^*J*_HH_ = 3.3 Hz). Additionally, a signal for bound ^15^NO_2_^–^ appears at δ_N_ 476.1. Hence, there is a strong hydride-^15^N coupling in this material that would be commensurate with SABRE. Subsequently, this sample was warmed to 298 K for 20 min. This led to the formation of multiple hydride-containing products, some of which display PHIP on exposure to *p*-H_2_ (see the Supporting Information). Pleasingly, a hyperpolarized signal for free Na^15^NO_2_ is observed at 298 K in the ^15^N NMR spectrum after SABRE transfer at −5 mG; a field in the mG range will be needed for efficient SABRE transfer.^51,55,56^ The resulting ^15^N signal enhancement was 134-fold and confirms reversible binding of NO_2_^–^. Unfortunately, when this sample was left at room temperature for >2 h, SABRE activity was lost because of catalyst decomposition. Hence, we sought to create alternative catalysts that would not only improve the ^15^N signal enhancement level but also be suitable for repeated measurement over long periods. Coligands have been used to achieve stability in conjunction with weakly binding substrate,^40,41,73^ to reduce spin dilution^42,75,76^ and to create hydride ligand chemical, rather than magnetic, inequivalence.^77^ Hence, we chose to follow this path as it offers a multitude of benefits to SABRE.

Thus, a sample containing [IrCl(COD)(IMes)] (**1**, 5 mM), Na^15^NO_2_ (5 equiv), and pyridine (3 equiv) was investigated by NMR spectroscopy. The initial formation of [Ir(^15^NO_2_)(COD)(IMes)] (**2**) was indicated. Clearly, nitrite outcompetes pyridine for the [Ir(COD)(IMes)]^+^ center. Subsequently, exposing this sample to 3 bar H_2_ at 254 K led again to the formation of neutral **3_._** Characterization data are provided in the Supporting Information. We note that known [Ir(H)_2_(IMes)(η^1^-COD)(pyridine)_2_]Cl (**4_A_**) forms alongside **3** in a 1:4 ratio. After warming the sample for 1 h at room temperature, further reaction to form two additional hydride-containing products takes place. Of these, [Ir(H)_2_(^15^NO_2_)(IMes)(pyridine)_2_] (**5_A_**), with characteristic hydride peaks at δ_H_ −21.24 and −22.45, dominates. The former resonance exhibits a ^2^*J*_NH_ splitting of 29 Hz, and both show ^2^*J*_HH_ couplings of −8 Hz.

A further minor product formed in this reaction proved to be Na[Ir(H)_2_(^15^NO_2_)_2_(IMes)(pyridine)] (**6_A_**). It yields hydride resonances at δ_H_ −22.02 (^2^*J*_NH_ = 29 Hz) and −23.01 with mutual ^2^*J*_HH_ splittings of −7 Hz. Interestingly, no evidence for the formation of *tris* pyridine-containing [Ir(H)_2_(IMes)(pyridine)_3_]Cl was observed^78^ and unlike the complexes formed in the absence of pyridine, pyridine-derived **5_A_** and **6_A_** proved stable when left at room temperature for >24 h. Given this stability, these species were suitable probes for rigorous assessment of their SABRE performance. When the ratio of Na^15^NO_2_ to pyridine was set to 5:3, the ratio of **5_A_** to **6_A_** in solution proved to be 85:15. Further addition of Na^15^NO_2_ (25 equiv), while maintaining the pyridine concentration, only moderately shifted the equilibrium between **5_A_** and **6_A_** to 80:20 thereby confirming that neutral [Ir(H)_2_(^15^NO_2_)(IMes)(pyridine)_2_] (**5_A_**) is the most thermodynamically stable of these products.

### SABRE Assessment of [Ir(H)_2_(^15^NO_2_)(IMes)(A)_2_] (**5_A_**) and Na[Ir(H)_2_(^15^NO_2_)_2_(IMes)(A)] (**6_A_**) Activity

In order for effective SABRE, the lifetime of the active catalyst must match with the propagating couplings and a level anticrossing condition should be met.^43,44,46^ To assess the SABRE performance of **5_A_** and **6_A_**, a series of shake and drop measurements were undertaken using a mu-metal shield to attenuate the Earth’s field by a factor of 300 to bring it into the range needed for efficient transfer. These measurements involved first exposing an NMR tube equipped with a *J*. Youngs Tap containing a solution of [IrCl(COD)(IMes)] (**1**, 5 mM), Na^15^NO_2_ (5 equiv), and pyridine (4 equiv) in methanol-*d*_4_ (0.6 mL) to H_2_ (3 bar) for 1 h to form an 85:15 ratio of **5_A_** to **6_A_** in solution. Subsequently, the H_2_ atmosphere was replaced with *p*-H_2_ (3 bar), and the sample was shaken for 10 s inside the mu-metal shield. After shaking, the sample was transferred into the 9.4 T detection field and an ^15^N NMR spectrum was recorded immediately.

Spectral analysis revealed that the free ^15^N signal of Na^15^NO_2_ was now ∼880-fold larger than that of the corresponding thermally polarized NMR spectrum, corresponding to a 0.29% ^15^N polarization level (Figure 1b). SABRE transfer to the ^15^N of unlabeled pyridine was also observed, and a 172-fold signal gain was quantified for its resonance at δ_N_ 301. ^15^N NMR signals for coordinated NO_2_^–^ ligands were also readily visible at δ_N_ 511.28 (*J*_HN_ = 29 Hz) for **5_A_** and at δ_N_ 509.7 (*J*_HN_ = 29 Hz) for the NO_2_^–^ in the equatorial position and at δ_N_ 483.7 for the ligand in the axial position of **6_A_**. Repeating the experiment after polarization transfer at 70 G and subsequently recording a ^1^H NMR spectrum revealed that PHIP-enhanced hydride resonances for **5_A_** and **6_A_**. SABRE hyperpolarization was also quantified for the ^1^H resonances of free pyridine as ∼230, 60, and 150-fold for its *ortho*, *meta*, and *para* positions, respectively, after 60 G transfer. No evidence for a PHIP-enhanced hydride resonance for [Ir(H)_2_(IMes)(py)_3_]Cl at δ_H_ −22.7 was observed. We conclude therefore that ^15^NO_2_^–^ sensitization is possible through the action of this coligand-supported catalyst.

### Effect of Polarization Transfer Field on the Level of ^15^N NMR Signal Gain in Na^15^NO_2_

To improve the levels of signal gain, a more precise polarization transfer field needs to be used. To investigate this effect, a sample containing [IrCl(COD)(IMes)] (**1**, 5 mM), Na^15^NO_2_ (5 equiv), and pyridine (4 equiv) in methanol-*d*_4_ (0.6 mL) was exposed to *p*-H_2_ (3 bar), and polarization transfer fields from +10 to −10 mG were deployed; these were created by a solenoid located within an mu-metal shield. A profile of the resulting SABRE enhanced resonance for Na^15^NO_2_ is presented in the Supporting Information. The highest signal enhancements were observed when the polarization transfer field was nominally +5 or −3 mG with gains of 1948 and 2054-fold, respectively. More precise probing of the polarization transfer field around these maxima revealed that improvement could be achieved using a −3.5 mG value. At this polarization transfer field, a 2329-fold signal gain was quantified through subsequent measurement at 9.4 T which corresponds to an ^15^N polarization level of 0.77%.

### Effect of the Coligand on SABRE Catalysis

**Figure 2:**
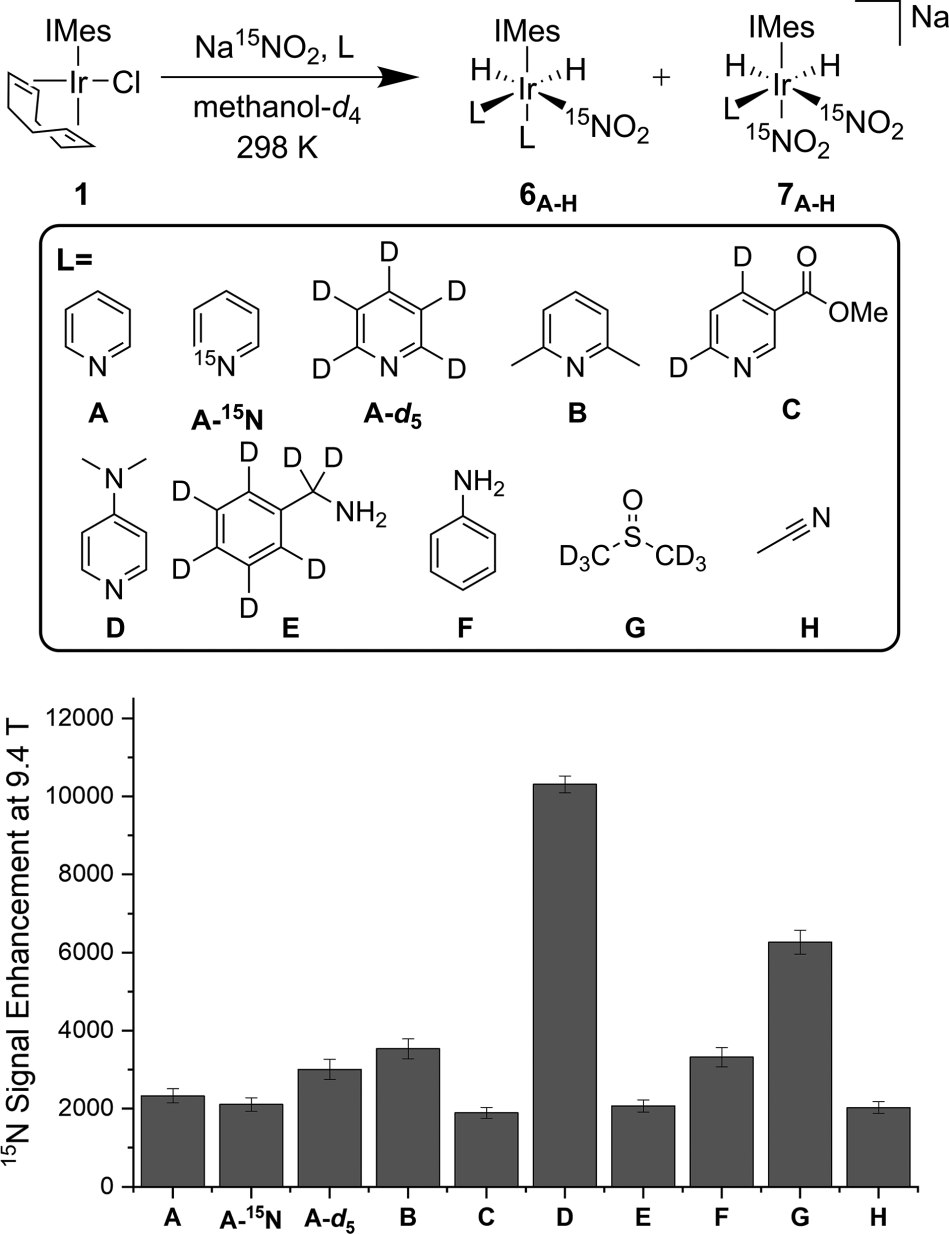
Graphical representation of the effect the coligand, **A**-**H**, has on the resulting SABRE polarization efficiency for Na^15^NO_2_ based on the precatalyst [IrCl(COD)(IMes)], Na^15^NO_2_ (25 equiv), and coligand (4 equiv) in methanol-*d*_4_ at 298 K. The ^15^N NMR signal enhancements occur after polarization transfer from 3 bar *p*-H_2_ in a −3.5 mG field.

To form **5_A_** and **6_A_**, ^15^NO_2_^–^ must out-bind the stabilizing coligand pyridine. Consequently, we hypothesized that the SABRE processes will be sensitive to the identity of this coligand; such a behavior has been observed previously during the SABRE polarization of sodium pyruvate by sulfoxides,^39^ and there are other examples.^41,72,79^ Additionally, isotopic labeling of these coligands, which reduces the number of polarization-acceptor spins at the metal center, has proven beneficial while additionally attenuating the effect of relaxation.^42,80,81^ A suitable range of coligands were therefore examined to test if it were possible to improve the measured polarization levels in free Na^15^NO_2_, as detailed in Figure 2.

In each case, samples containing [IrCl(COD)(IMes)], Na^15^NO_2_ (25 equiv), and the coligand (**A**-**H**, 4 equiv) were prepared and then exposed to 3 bar H_2_ at 298 K for 1 h to form the corresponding complexes **6** and **7**. Subsequently, a sample was exposed to 3 bar *p*-H_2_, in a −3.5 mG field, prior to its rapid insertion into the 9.4 T detection field. The second coligand tested was ^15^N labeled pyridine (**A-^15^N**), and this reduced the signal enhancement level for Na^15^NO_2_ from 2329-fold to 2107-fold. This is likely to reflect the increase in spin dilution associated by increasing the proportion of spin-1/2 nuclei that can accept polarization. The resonance for free ^15^N-pyridine at δ_N_ 301 now exhibits a signal gain of 1558-fold. In contrast, the use of pyridine-*d*_5_ (**A-*d*_5_**) improves the SABRE hyperpolarization for Na^15^NO_2_ as the new enhancement level increases to 3007-fold. As expected, all the pyridine isotopologues yield analogous complexes, **5** to **6**, in a common 85:15 ratio. Hence, catalyst speciation is constant and the ca. 30% improvement, compared to undeuterated **A**, likely reflects both slower relaxation in the active catalyst and reduced polarization.

To further modulate the coligand, other pyridyl derivatives having different steric and electronic properties were examined. Recently reported 2,6-lutidine (**B**)^82,83^ was chosen as its *ortho* methyl groups hinder binding to the metal center, a change which might promote ligand loss. When **B** is employed with Na^15^NO_2_, an increase in the SABRE polarization level is indeed observed when compared to pyridine. Interestingly, the ratio of **5_B_** to **6_B_** has changed to 95:5. However, slow activation means that **3** (c.f. Figure 1a) is still visible after 1 h at room temperature; at this stage, it exists in a 1:1 ratio with **5_B_**. Unfortunately, when this sample is left under a 3 bar atmosphere of H_2_ for a longer time to allow full activation, sample degradation and the formation of multiple hydride-containing complexes are noted. Hence, **B** fails to provide the stability needed.

The use of electron-deficient methyl 4,6-*d*_2_-nicotinate (**C**), which has been shown to exhibit ^1^H polarization levels of ca. 60% itself under SABRE,^42,60^ as a coligand was found to decrease the signal enhancement of Na^15^NO_2_ to 1894. The formation of mono-**C**-substituted **6_C_** is favored by this change as the ratio of **5_C_** to **6_C_** became 1:2. In contrast, electron-rich dimethylamino pyridine (**D**, DMAP) forms **5_D_** in a 17:1 ratio with **6_D._** Consequently, electron-donating coligands favor the formation of a *bis*-co-ligand substituted complex. Additionally, a significantly improved 10,313-fold ^15^N signal enhancement is now observed for Na^15^NO_2_ which corresponds to the creation of a 3.4% ^15^N polarization level.

Nonheterocyclic ligands can also be utilized for SABRE. As such, amine ligands have been shown to be able to form stable SABRE catalysts and are effective agents for SABRE-relay polarization transfer.^80,81,84−86^ When utilized as a coligand for the hyperpolarization of Na^15^NO_2_, benzylamine-*d*_7_ (**E-*d*_7_**) led to a ^15^N signal gain of 2070-fold. The two hydride-containing complexes **5_E_** and **6_E_** were formed under these conditions in a ca. 1:1 ratio with hydride resonances at δ_H_ −22.10 and −23.40 and δ_H_ −22.36 and −22.72, respectively. When aniline (**F**) was used as the coligand, a 3322-fold signal gain for Na^15^NO_2_ was quantified. In this sample, **5_F_** now dominates.

Similarly, sulfoxides have proven to be efficacious for the hyperpolarization of sodium pyruvate and weakly coordinating substrates.^39,40,79,87^ The coligand DMSO-*d*_6_ (**G**) gave a 6270-fold signal enhancement for Na^15^NO_2_. Interestingly, while Na[Ir(H)_2_(^15^NO_2_)_2_(IMes)(DMSO-*d*_6_)] (**6_G_**) is now dominant, a second isomer **7_G_**, where the two ^15^NO_2_ ligands lie *cis* to one another and *trans* to hydride is observed. This complex gives rise to a single hydride resonance at δ_H_ −22.32 where *J*_NH*cis*_ + *J*_NH*trans*_ is 27.6 Hz. The ^15^NO_2_ resonance of **7_G_** appears at δ_H_ 502. Isomer **5_G_** is also detected, but now as a minor species, with the ratio of **5_G_**:**6_G_**:**7_G_** in solution being ∼1:9:5. Characterization data for these complexes are provided in the Supporting Information. Finally, acetonitrile^88^ gave a 2029-fold ^15^N signal gain for NO_2_^–^. For acetonitrile, the neutral complex [Ir(H)_2_(^15^NO_2_)(IMes)(acetonitrile)_2_] (**5_H_**), with hydride resonances at δ_H_ −22.66 (^2^*J*_NH_ = 26.7 Hz, ^2^*J*_HH_ = −7 Hz) and −21.77 (^2^*J*_HH_ = −7 Hz), was the only complex observed. Clearly substantial coligand effects occur, with **D** proving optimal.

### Identifying the Optimum DMAP (**D**):Na^15^NO_2_ Ratio

Interestingly, this ligand yielded the highest concentration of isomer **5**. We postulated that the concentration of **5_D_** in solution could be further manipulated by changing the number of equivalents of **D** in relation to [IrCl(COD)(IMes)] (**1**) and Na^15^NO_2_. Therefore, a series of samples were prepared with between 3 and 20 equiv of **D** relative to **1**. After activation, they were exposed to 3 bar *p*-H_2_ while located in a −3.5 mG polarization transfer field. The resulting signal enhancements at 9.4 T are shown in the Supporting Information.

When three equivalents of **D** (with respect to iridium) are utilized, a 9086-fold signal enhancement is observed with the corresponding **5_D_**:**6_D_** ratio being 8:1. Increasing the concentration of DMAP to four equivalents improved the signal gain seen at 9.4 T to 11,019-fold. The ratio of complex **5_D_**:**6_D_** also increased to 17:1. Further incremental increases in DMAP concentration, to 6, 8, and 10 equiv, gave signal enhancements of 12,036, 12,079, and 11,888-fold, respectively. The ratio **5_D_**:**6_D_** was now 24:1 in all three samples. At higher loadings of **D**, the formation of [Ir(H)_2_(IMes)(**D**)_3_]Cl is observed, as a single hydride resonance at δ_H_ −23.00. Clearly, this catalyst does not transfer hyperpolarization to Na^15^NO_2_ and hinders the overall ^15^N signal gain because of consumption of *p*-H_2_. Therefore, we conclude that a sensible DMAP level lies between 6 and 10 equivalents with respect to iridium. This creates SABRE beneficial **5_D_** as the dominant species in solution.

### Synthesis and Utilization of DMAP-*d*_2_ for SABRE

As stated, deuteration of ligands within the active catalyst can be beneficial.^42,70,76,77,88^ We postulated that deuteration of the *ortho* protons in **D**, to give DMAP-*d*_2_, may lead to further improvements in ^15^N polarization. Thus, DMAP-*d*_2_ was synthesized via H/D exchange from DMAP in D_2_O under microwave irradiation as reported in the literature.^89^ Examination of a sample containing [IrCl(COD)(IMes)] (5 mM), DMAP-*d*_2_ (6 equiv), and Na^15^NO_2_ (25 equiv) in methanol-*d*_4_ and exposure to 3 bar *p*-H_2_ at a polarization transfer at −3.5 mG led to a signal gain of 13,811 after investigation at 9.4 T (4.56% ^15^N polarization level). The corresponding value with ^1^H-DMAP was 12,036, and hence introducing the ^2^H is beneficial to SABRE.

### Effect of NHC Identity on the Efficiency of Na^15^NO_2_ Polarization

**Figure 3:**
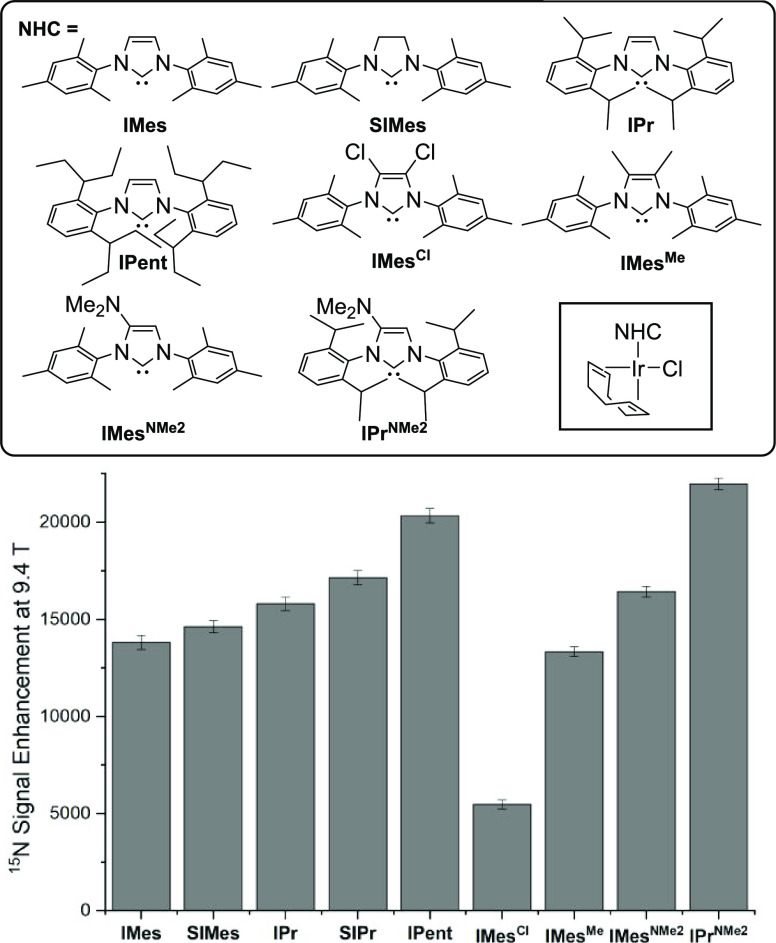
Effect of NHC on the ^15^N NMR signal gain for Na^15^NO_2_ with precatalyst [IrCl(COD)(NHC)] (5 mM), DMAP-*d*_2_ (6 equiv), and Na^15^NO_2_ (25 equiv) in methanol-*d*_4_ after polarization transfer at −3.5 mG under 3 bar *p*-H_2_.

Aiming to improve the polarization outcome still further, a study of the effect of the NHC ligand was completed in conjunction with DMAP-*d*_2_. Previously, we have shown how manipulation of the steric and electronic properties of this ancillary ligand can result in improved ^1^H, ^13^C, and ^15^N signal enhancements because of changes in the rate of ligand exchange.^60^ We sequentially increased the steric bulk of the NHC (quantified by the magnitude of %BurV^90,91^) to drive ligand exchange (Figure 3). On moving from the IMes ligand (%BurV = 31.2) to SIMes (32.7), we saw a 14,628-fold signal gain which is a slight improvement from the 13,811-fold signal gain previously observed for IMes. IPr (33.6) and SIPr (35.7) both also led to increased signal enhancements of 15,799 and 17,149-fold. However, the best result was obtained for IPent as a 20,337-fold signal gain which has the highest %BurV of 43.4.

Next our focus turned to the electronic properties of the NHC ligand (Figure 3). As expected, electron-deficient IMes^Cl^, which has chloro substituents on the imidazole ring, reduced the signal enhancement to just 5471. Introducing methyl groups on the imidazole ring showed a minimal effect when compared to IMes (13,336-fold vs 13,811-fold, respectively). However, introduction of a single −NMe_2_ group increased the signal enhancement level to 16,427-fold at 9.4 T.

To combine the steric and electronic effects, we utilized the ligand IPr^NMe2^, which has previously proven to be effective for Buchwald–Hartwig amination catalysis,^92,93^ to form the precatalyst [IrCl(COD)(IPr^NMe2^)]. This catalyst system gave the highest ^15^N signal enhancement for free Na^15^NO_2_ seen, 21,967-fold at 9.4 T which is equivalent to 7.2% polarization.

### Effect of Na^15^NO_2_ Concentration on Signal Enhancement

A series of samples were prepared which contained varying excesses of Na^15^NO_2_ relative to 5 mM of [IrCl(COD)(IPr^NMe2^)] and a constant 30 mM of DMAP-*d*_2_. The corresponding hyperpolarization results are presented in the Supporting Information. Reducing the substrate excess to 10 equiv with respect to iridium increased the ^15^N signal enhancement to 36,629-fold, but for just 4 equiv a value of 62,470-fold was found which is equivalent to 20.6% ^15^N polarization. Conversely, increasing the substrate loading to 50 equiv reduced the signal gain to 10,382 (3.17%), albeit with an improved signal to noise ratio. When the concentration of [IrCl(COD)(IPr^NMe2^)] is reduced to 0.25 mM, while maintaining the 1:4 molar ratio with Na^15^NO_2_, the ^15^N polarization level increases to 28.42%. This phenomenon is likely the result of increasing the excess of *p*-H_2_, the limiting reagent, relative to this substrate.^53,60^

### Detection of Unlabeled NaNO_2_

Given the high signal gains obtained for Na^15^NO_2_, we tested a sample where the ^15^N label was present at natural abundance. This sample contained 20 mM of NaNO_2_ and was examined with [IrCl(COD)(IPr^NMe2^)] (5 mM) and DMAP-*d*_2_ (6 equiv,). An ^15^N signal was easily seen whose signal enhancement was 115,592-fold at 9.4 T; this corresponds to a 38.1% polarization level.

### Effect of 15-Crown-5 and Alternative Solvents

Unfortunately, the ionic nature of NaNO_2_ acts to limit its solubility in the range of organic solvents that are typically employed for SABRE catalysis; it has a moderate solubility in methanol; however, it is sparingly soluble in other primary alcohols and insoluble in most apolar solvents. In an attempt to increase methanol-*d*_4_ solubility, the macrocycle 15-crown-5 was added, which has a high chelating affinity for Na^+^.^94,95^ SABRE transfer was therefore undertaken on a sample containing [IrCl(COD)(IMes)] (5 mM), Na^15^NO_2_ (25 equiv), DMAP (6 equiv), and 15-crown-5 (25 equiv) in methanol-*d*_4_. This led to an ^15^N signal enhancement of 12,044-fold at 9.4 T for ^15^NO_2_^–^ which corresponds to 3.97% and reflects a 10% improvement over the analogous measurement with no 15-crown-5. Interestingly, the ratio of **5_D_** to **6_D_** in solution was 99:1, as opposed to 91:9 seen in the absence of 15-crown-5. For the optimized catalyst and coligand ([IrCl(COD)(IPr^NMe2^)] (5 mM), Na^15^NO_2_ (25 equiv), and DMAP-*d*_2_ (6 equiv)), the effect of 15-crown-5 proved to be less pronounced, with the ^15^N signal gain improving from 21,967-fold to just 23,114-fold. Hence, solvent effects on this catalysis are substantial and likely to change ligand exchange rates in addition to catalyst speciation.

When an NMR sample containing [IrCl(COD)(IMes)] (5 mM), Na^15^NO_2_ (25 equiv), and **D** (6 equiv) was prepared in dichloromethane-*d*_2_ (0.6 mL), the impact of insolubility of Na^15^NO_2_ was immediately evident. After sonication for 30 min, the sample was exposed to H_2_ (3 bar). Investigation by NMR spectroscopy revealed just [Ir(H)_2_Cl(IMes)(DMAP)_2_]. However, when an analogous sample was prepared containing 15-crown-5 in a 1:1 ratio with Na^15^NO_2_, a different hydride-containing complex formed. Its hydride resonances appear at δ_H_ −22.66 (^2^*J*_HN_ = 27.5 Hz and ^2^*J*_HH_ = −7 Hz) and δ −23.00 (^2^*J*_HH_ = −7 Hz) and match those of **5_D_**. After SABRE transfer in a −3.5 mG field, a 3586-fold signal enhancement was observed at 9.4 T for the free ^15^NO_2_^–^ resonance at δ_N_ 618. Warming this sample to 308 K prior to polarization transfer significantly improved the signal gain to 7248-fold and indicates that slow ligand exchange limits the polarization level attained. However, warming further to 323 K yielded no further increase. Using the electron-rich and sterically encumbered precatalyst [IrCl(COD)(IPr^NMe2^)] also yielded improved polarization transfer as an 8149-fold signal gain is seen at 9.4 T. Warming this sample, however, had no benefit. Hence, we have demonstrated how significant polarization levels for ^15^NO_2_^–^ result in dichloromethane-*d*_2_ if 15-crown-5 is present.

### Assessment of Na^15^NO_2_ Relaxation Rates

DNP hyperpolarized Na^15^NO_2_ is reported to have a *T*_1_ of 14.8 s in D_2_O at 5.8 T.^15^ We used a low-tip angle approach to assess the *T*_1_ of this SABRE-polarized product at 9.4 T. It was found to be comparable at 16.45 s in the presence of a SABRE catalyst. This value was also determined using an automated hyperpolarization device under reversible flow,^96^ after first conducting the SABRE process at −3.5 mG, prior to turning off the *p*-H_2_ supply and holding the sample in a defined magnetic field for a period of time, prior to transfer to 9.4 T to acquire a spectrum. Repeating this process for a number of time points enables the effective low field *T*_1_ value to be calculated. This analysis was undertaken on samples that were stored in the mu-metal shield (ca. 300-fold shielding) or at −3.5 mT. The new *T*_1_ values were 14.9 and 11.2 s, respectively (see the Supporting Information). These values suggest that there will be sufficient time to use the hyperpolarized ^15^NO_2_^–^ resulting from SABRE synthetically to create other hyperpolarized products as 3 × *T*_1_ is available before a signal vanishes. Interestingly, as the *T*_1_ values for ^15^N nuclei can dramatically be extended when they are located in an appropriate magnetic field, accessing reaction times of many minutes may be possible.^55,97,98^ We are currently exploring this, but here we show how rapid reactions can be evaluated through ^15^N NMR at high field is detailed in the following sections.

### Conversion of Hyperpolarized Na^15^NO_2_ to a Diazonium via NO^+^

The first reaction we consider is the important Sandmeyer reaction that rapidly converts arylamines into arylhalides via a diazonium salt intermediate.^99^ Since the first reported example in 1884,^100^ it has become a mainstay of organic chemistry and many related reactions have been discovered.^101^ Classically, it utilizes either stoichiometric or catalytic amounts of a copper halide, although a number of metal free variants are known.^102−104^ The formation of the diazonium salt intermediate proceeds via nitrous acid addition, which is formed in situ from the reaction of NaNO_2_ and a strong acid. We sought to follow a diazotization reaction by ^15^N hyperpolarized NMR spectroscopy. To do this, we first created a solution of hyperpolarized Na^15^NO_2_ using the previously optimized conditions ([IrCl(COD)(IPr^NMe2^)] (5 mM), DMAP (30 mM), Na^15^NO_2_ (125 mM) in methanol-*d*_4_ (0.6 mL)). A solution of aniline (150 mM) and conc. HCl (100 μL) in methanol-*d*_4_ (100 μL) was then added, and the resulting NMR tube was immediately transferred into the spectrometer and investigated using a *T*_1_-corrected variable flip angle pulse sequence. It took between 3 and 5 s to start this series of measurements, and the resulting hyperpolarized signals were indicative of nitrous acid (H^15^NO_2_, δ_N_ 563) forming phenyl diazonium chloride (δ_N_ 314) and *ortho*-^15^N_2_ (δ_N_ 308). Their identity was confirmed by their independent synthesis and comparison to literature data.^105^ Over the course of 30 s, the response for H^15^NO_2_ vanished.

**Figure 4:**
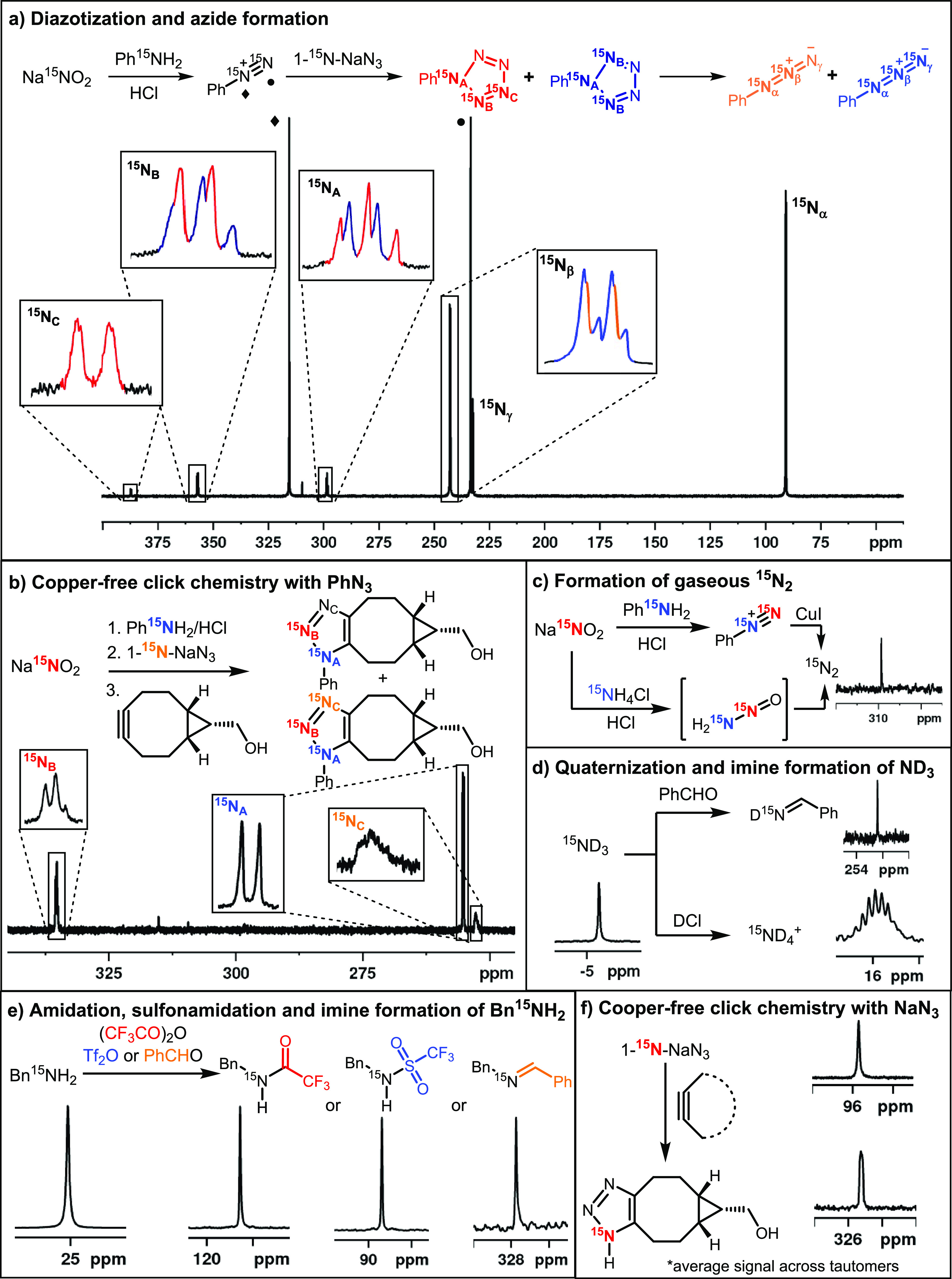
Establishing SABRE hyperpolarization allows the reactivity of ^15^N-containing synthons to be assessed. (a) Multistep reaction from Na^15^NO_2_ that tracks diazotization with aniline-^15^N and the subsequent formation of two isotopomers of phenyl azide after reaction with 1-^15^N-NaN_3_ in a process that proceeds through a cyclic intermediate; (b) copper-free click reaction of PhN_3_ formed as in part (a); (c) formation of N_2_(g); (d) ND_3_ quaternization with DCl(aq) and imine formation with benzaldehyde; (e) amidation, sulfonamidation, and imine formation of benzylamine-^15^N; (f) copper-free click reaction of 1-^15^N-NaN_3_.

When this process was repeated with aniline-^15^N, the reaction monitoring step revealed the detection of hyperpolarized responses for both of the ^15^N centers in the diazo product at δ_N_ 315.0 and 232.6 in agreement with the literature (Figure 4a).^106^ The hyperpolarization of both of the ^15^N sites happens even though aniline itself was not hyperpolarized. Consequently, efficient polarization transfer between them takes place during their time as a coupled spin pair at low field. As a control, we exposed a sample of phenyl diazonium chloride and the catalyst to *p*-H_2_ at −3.5 mG and noted no hyperpolarized ^15^N resonance result. Consequently, all the hyperpolarized signals seen during this reaction originate from the initially hyperpolarized Na^15^NO_2_ synthon.

Figure S12 of the Supporting Information details how the hyperpolarization level of unlabeled NaNO_2_ is sufficient to allow the detection of phenyl diazonium chloride without the need for isotopic labeling.

### Reactions of Hyperpolarized ^15^N_2_-Phenyl Diazonium Chloride

Phenyl diazonium chloride proved to have hyperpolarized *T*_1_ values for ^15^N_1_ and ^15^N_2_ of 29.4 and 39.2 s, respectively, at 9.4 T. Additionally, it proved to be relatively stable under these conditions as only limited decomposition to hyperpolarized ^15^N_2(g)_ (δ_N_ 308) was seen. This meant that we could explore the reactivity of this diazonium salt in situ. It is known that such salts liberate N_2_ under photochemical or transition-metal-catalyzed processes.^107,108^ Under our hyperpolarized regime, addition of CuI saw its rapid conversion into N_2_ and consequently a strong signal was seen at δ_N_ 308.

A similar hyperpolarized diazonium salt solution was prepared and then treated with NaN_3_ to examine the formation of phenyl azide. Rapid monitoring enabled the collection of a hyperpolarized ^15^N NMR spectrum with strong resonances at δ_N_ 242.2 and 90.1 that share a common ^2^*J*_NN_ of 13.8 Hz because of this species. According to the literature, this reaction could proceed via a cyclic and/or acyclic intermediate, species which would deliver five and three distinct ^15^N signals, respectively.^109−111^ Interestingly, we detect transient signals at δ_N_ 356.8 and 298.2 (both with ^2^*J*_NN_ = 16.7 Hz) for the site connected to the C_6_H_5_ ring which we assign to this product. Upon repeating this study with 1-^15^N NaN_3_, these two signals gain further complexity and appear alongside one other at δ_N_ 387.3 (d, 17 Hz). These additional features are reflective of the two possible isotopologues that can result from ^15^N_1_–N_3_^–^ addition to form a cyclic intermediate, which place a Ph–^15^N next to two chemically equivalent ^15^N groups (a triplet at δ_N_ 298.6 of 17 Hz is seen for it alongside a doublet of 17 Hz at δ_N_ 356.9) or one (a doublet at δ_N_ 298.6 of 17 Hz is now seen) alongside a further triplet at 356.9 of 17 Hz and a doublet at δ_N_ 387.3 (d 17 Hz) for the next and more remote center (Figure 4a) ^15^N of the N_5_ ring. Hence, all three unique signals for this cyclic intermediate have been detected. We note that its conversion into phenyl azide (Ph–^15^N=^15^N^+^=^15^N^–^ and Ph–^15^N=^15^N^+^=N^–^) proceeds rapidly at 298 K, and the signals for this product also appear, δ_N_ 90.3, 242.5, and 232, with apparent *T*_1_ values of 56, 192, and 101 s at 9.4 T, all respectively.

These long *T*_1_ values enable the creation of strongly hyperpolarized phenyl azide. When the reactive alkyne, (1*R*,8*S*,9*s*)-bicyclo[6.1.0]non-4-yn-9-ylmethanol,^112^ is added to this hyperpolarized sample in a third synthetic step, further reaction to form the corresponding triazole occurs. Despite the corresponding ^15^N signal’s *T*_1_ in this product proving to be <10 s, its formation is readily indicated in the associated hyperpolarized ^15^N NMR measurements through three signals at δ_N_ 335.4, 255.3, and 252.7 that can be linked through mutual ^2^*J*_NN_ couplings of 12.8 Hz (Figure 4b). As copper-free click chemistry is used widely for bioconjugation with nuclei acids, we expect such measurements to help in the optimization of pharmaceutical preparations and/or in vivo detection.^113,114^

These data have clearly illustrated the successful examination of a multistep reaction as it proved possible to simultaneously see ^15^N signals for the phenyl diazonium salt, the pentazole intermediate and phenyl azide (Figure 4a) or the pentazole intermediate, phenyl azide, and the triazole (Figure 4b). We plan to develop methods to extract precise kinetic data for these changes in the future.

### Conversion of Hyperpolarized Na^15^NO_2_ to ^15^N_2_ through Reaction with ^15^NH_4_Cl/HCl

N_2_ gas spontaneously forms from the diazonium salts of primary amines. Consequently, as ^15^NH_4_Cl is readily available we monitored its reaction with Na^15^NO_2_ and saw strong signals for ^15^N_2_ in solution (see Figure 4c). We have therefore detailed two facile approaches to hyperpolarized ^15^N_2_, we are currently exploring as routes to potentially important *p*-N_2_.^54,74^

### Utilization of ^15^N Hyperpolarized Azide, Amines, and Ammonia as Probes of Reactivity

The SABRE hyperpolarization of NH_3_ and amines, such as benzylamine, and their use in SABRE-relay have been extensively reported.^80,81,84−86^ Additionally, ammonia and amines have been used as a coligand that leads to improved SABRE catalysis.^62,115^ The ^15^N polarization of benzylamine-^15^N (**E-^15^N**) is reported to be ca. 800-fold at 9.4 T.^84^ This involves the action of [Ir(H)_2_(IMes)(**E-^15^N**)_3_]Cl in dichloromethane-*d*_2_ solution. We restudied this process to improve the SABRE outcome and thereby provide access to a further functional group to demonstrate hyperpolarized reactivity screening. Using the same conditions as previously reported ([IrCl(COD)(IMes)] (5 mM) and benzylamine-^15^N (**E-^15^N**, 7 equiv)), we determined that optimal SABRE transfer occurs at −4 mG. At this transfer field, a 7751-fold signal enhancement was achieved at 9.4 T. As the rate of benzylamine dissociation from [Ir(H)_2_(IMes)(**E-^15^N**)_3_]Cl in dichloromethane-*d*_2_ is slow,^45,85^ we found that warming the sample to 308 K further improved the enhancement level to 11,211-fold which corresponds to 3.7% ^15^N polarization; it has 14 s *T*_1_ at 9.4 T in the absence of the catalyst and 12.8 s when it is present. We predict that further optimizations could improve this value; however, the resulting signal strengths are sufficient to explore its reactivity. We exemplify now the utilization of hyperpolarized **E-^15^N** as a synthon for amidation, sulfonamidation, and imine formation. This resulted in the ^15^N detection of the products shown in Figure 4e. Their identity was verified by independent synthesis as described in the Supporting Information or by comparison to literature data. In particular, the addition of trifluoroacetic anhydride to hyperpolarized **E-^15^N** led to the formation and detection of *N*-benzyl-trifluoroacetamide-^15^N in the resulting ^15^N NMR spectrum through a signal at δ_N_ 116.4. Similarly, triflic anhydride reacted to yield the analogous sulfonamide with a resonance at δ_N_ 88.6. Finally, addition of benzaldehyde to **E-^15^N** produced the imine condensation product as evident from a peak at δ_N_ 327.7.

Ammonia is also widely used in synthetic chemistry and we sought to test whether its reactivity could be probed while hyperpolarized. As gaseous ^15^NH_3_ was expensive and difficult to handle, we used an alternative ammonia source.

This involved taking a 1:1 mixture of ^15^NH_4_Cl/KO^*t*^Bu and adding it to **1** in the presence of H_2_ with the result that [Ir(H)_2_(IMes)(^15^ND_3_)_3_]Cl forms in methanol-*d*_4_. After polarization transfer at −4 mG, a 3268-fold ^15^N signal gain was quantified for the free ^15^ND_3_ signal. However, over the course of ca. 1 h, the signal enhancement diminishes when the SABRE process is repeated. In contrast, the use of ^15^NH_4_OH (available as a 14 molar solution in H_2_O) yielded the same active catalyst, but the sample was now stable for >24 h. The ^15^N signal enhancement is also slightly improved to 3765-fold. Changing the NHC ligand proved to have a modest effect on SABRE efficacy (see the Supporting Information), and warming the sample derived from **1** to 308 K improved the signal gain to 4521-fold. However, dramatic improvements are observed with a coligand. While the coligands DMSO-*d*_6_, CD_3_CN, NO_2_^–^, and DMAP (see the Supporting Information) were explored, pyridine-*d*_5_ proved to give the highest signal gain of 15,145-fold (5.0% polarization). The ^15^N *T*_1_ value for ^15^ND_3_ at 9.4 T proved to be 37 s so there is again a wide time window over which a reaction can be examined. Protonation of ^15^ND_3_ with DCl in D_2_O led to the detection of hyperpolarized ^15^ND_4_^+^ as a signal δ_N_ 15.93 with resolved ^15^N-D scalar couplings, *J*_ND_, of 10.8 Hz and a hyperpolarized *T*_1_ of 33.6 s (Figure 4d).

The SABRE hyperpolarization of 1-^15^N NaN_3_ itself using the coligand strategy with DMAP and **1** also proved successful. The reaction was found to proceed to form [Ir(H)_2_(DMAP)_2_(IMes)(^15^N=N=N)] which exhibits hydride signals at δ_H_ −23.1 (^2^*J*_HH_ = 8 Hz) and δ_H_ −25.0 (^2^*J*_HH_ = 8 Hz and ^2^*J*_NH_ = 8 Hz) alongside [Ir(H)_2_(DMAP)_3_(IMes)]Cl (δ_H_ –22.8). SABRE transfer at −3.5 mG yielded 3.2% hyperpolarization of the N_3_^–^ signal at δ_N_ 95.7. A hyperpolarized solution of NaN_3_ was then reacted directly with (1*R*,8*S*,9*s*)-bicyclo[6.1.0]non-4-yn-9-ylmethanol to form the triazole. Under these conditions, a single hyperpolarized ^15^N response for the product was visible at δ_N_ 321.3 as expected and is shown in Figure 4f.

### Producing Hyperpolarized NO_2_^–^ in Water

**Figure 5:**
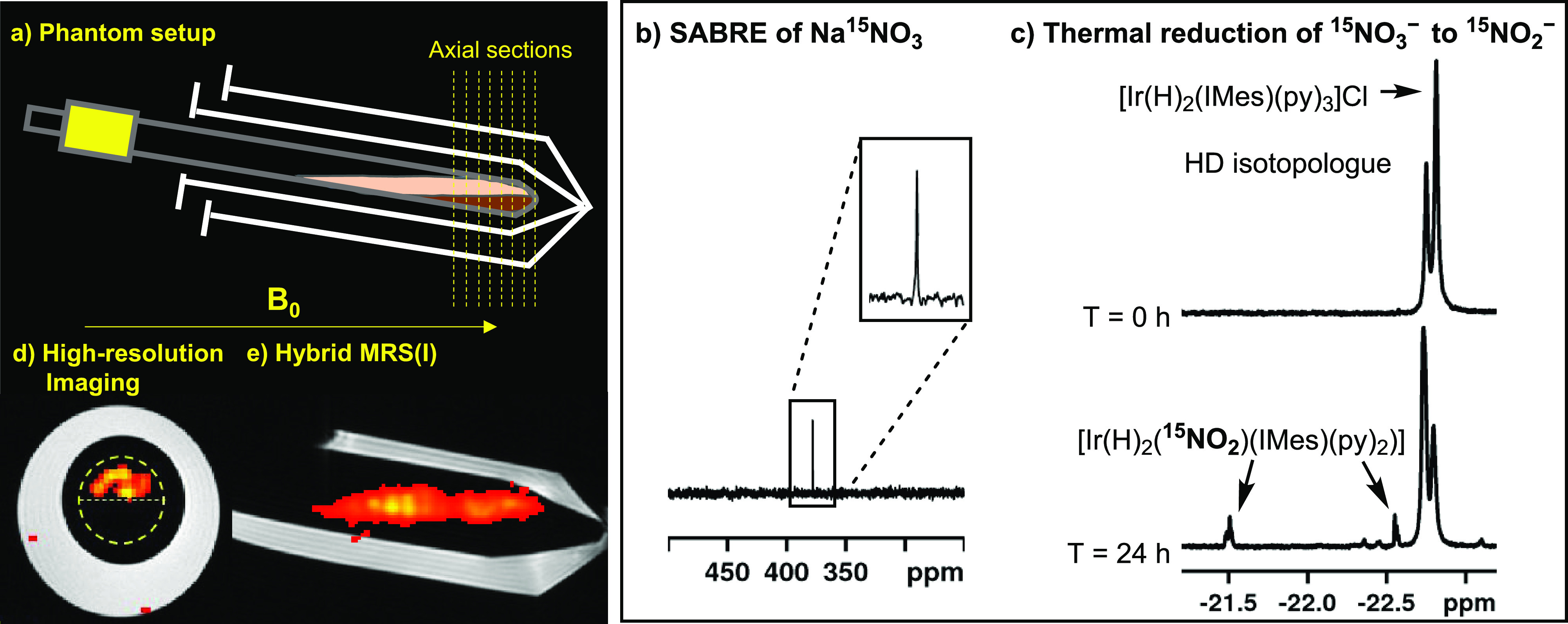
Establishing the imaging capability of SABRE hyperpolarized ^15^N O_2_^–^ in biocompatible solvents. (a) Setup for hyperpolarized imaging at 7 T of a reaction cell used for the biphasic preparation of ^15^NO_2_^–^; (b) SABRE hyperpolarized ^15^N NMR spectrum of Na^15^NO_3_ created with [Ir(COD)(IMes)(pyridine)]BF_4_, DMSO-*d*_6_ (2 equiv), Na^15^NO_3_ (25 equiv), and 3 bar *p*-H_2_ at 9.4 T; (c) ^1^H NMR spectra of the hydride region establish the conversion of NO_3_^–^ to NO_2_^–^ via the detection of Ir(H)(^15^NO_2_)(IMes)(py)_2_]; (d) high-resolution three-dimensional axial acquisition using spiral (out) encoding of 6 cm^3^ over a 64 × 64 × 8 matrix with a 6 s acquisition time; (e) hybrid MRS(I) data collected using an EPSI pulse sequence and a 64 × 64 matrix (512 spectral points).

For biological applications, it is desirable to produce hyperpolarized NO_2_^–^ in water. Unfortunately, **5_A_** did not form when the analogous reaction was conducted in this solvent. Preforming **5_D_** in methanol-*d*_4_ prior to removing the solvent and replacing it with D_2_O was also unsuccessful. One further way to achieve an aqueous bolus is to use a biphasic^116^ approach with dichloromethane-*d*_2,_ which could benefit from the fact that the catalyst is not present in the aqueous layer.^65,117^ A sample containing [IrCl(COD)(IMes)] (5 mM), Na^15^NO_2_ (25 equiv) and DMAP (6 equiv) and 15-crown-5 (25 equiv) in dichloromethane-*d*_2_ (0.3 mL) was prepared and exposed to H_2_ (3 bar) to form the active catalyst. D_2_O (0.3 mL) was then added under a nitrogen atmosphere. After SABRE transfer at −3.5 mG and phase separation, two hyperpolarized signals were seen in the corresponding ^15^N NMR spectrum for Na^15^NO_2_ at δ_N_ 618 and δ_N_ 609. These peaks had relative intensities of 1:70 and were assigned to Na^15^NO_2_ dissolved in the dichloromethane-*d*_2_ and D_2_O phases respectively by comparison with data from independent solutions. Assuming that all of the Na^15^NO_2_ was present, the D_2_O layer results in a ^15^N signal gain of 4667-fold. This will be an underestimate of the actual signal gain. As 15-crown-5 can also play a role as a phase-transfer catalyst,^118^ a further 25 equiv was added to the sample and this proved to increase the signal enhancement level to 13,794-fold. Further additions of 15-crown-5 did not improve on this; however, warming the sample to 308 K resulted in a 26,327-fold signal gain at 9.4 T. This is equivalent to an 8.69% polarization level in aqueous Na^15^NO_2_. Hence, we have created a simple route to hyperpolarized Na^15^NO_2_ in biocompatible water. The level of signal gain compares favorably with the <1% reported with DNP.^15^ To confirm the phase distribution of Na^15^NO_2_, a series of ^15^N MRI images were recorded on a 10 mm-diameter sample tube as detailed in the Supporting Information. The data in Figure 5 details these results which confirm both separation and the fact that the associated signal strengths are sufficient to allow for high-sensitivity ^15^N imaging of NO_2_^–^.

### SABRE Hyperpolarization of Na^15^NO_3_

In contrast to nitrite, nitrate is usually noncoordinating; however, there are examples of it functioning as a weak monodentate or bidentate ligand.^29,119−123^ To further explore SABRE’s use as a tool to polarize materials featuring in the nitrogen cycle, we explored the SABRE hyperpolarization of Na^15^NO_3_. As expected, in the absence of a coligand no active SABRE catalyst formed in the reaction between [IrCl(COD)(IMes)] and Na^15^NO_3_ under a H_2_ atmosphere (3 bar) in methanol-*d*_4_. We screened a number of coligands (DMAP, 2-picoline, DMSO-*d*_6_, DPSO, or CD_3_CN) and saw no evidence for the ^15^N polarization of Na^15^NO_3_. In each case, the dominant hydride-containing species in solution was [Ir(H)_2_(IMes)(coligand)_3_]Cl or [IrCl(H)_2_(IMes)(coligand)_2_]. However, when the ionic precatalyst [Ir(COD)(IMes)(pyridine)]BF_4_ was used with DMSO-*d*_6_ (2 equiv), a 547-fold signal enhancement for the ^15^NO_3_^–^ signal at 9.4 T (0.18% ^15^N polarization) is observed (Figure 5b). No direct evidence for an NO_3_^–^ containing complex could be found, and therefore, polarization transfer must occur through a very low concentration species.

### Unexpected Reduction of Sodium Nitrate

During the course of these investigations, a hyperpolarized ^15^N NMR signal at δ_N_ 511.28 (*J*_HN_ = 28.5 Hz) appears over the course of 0.5 h when pyridine alone is used as a coligand. This matches the equatorial NO_2_^–^ resonance previously observed for [Ir(H)_2_(^15^NO_2_)(IMes)(pyridine)_2_] (**5_A_**). Relatively strong polarized signals for free pyridine and the pyridine ligand *trans* to hydride in [Ir(H)_2_(IMes)(py)_3_]Cl (δ_N_ 299.6 and 255.7, respectively) were also observed in these NMR spectra. As expected, the corresponding ^1^H NMR spectrum is dominated by the hydride signal of [Ir(H)_2_(IMes)(py)_3_]Cl which appears at δ_H_ −22.7, although a weakly PHIP-enhanced signal for **5_A_** is visible in this spectrum at δ_H_ −21.49 (the peak at δ_H_ −22.71 cannot be observed because of overlap). No evidence for **6_A_** was observed in either the ^1^H or ^15^N NMR spectra which indicates that **5_A_** is likely to be the kinetic product of this reaction. After waiting for a further 1 h, refreshing the sample with *p*-H_2_, and repeating the SABRE process, a polarized signal for free Na^15^NO_2_ (δ_N_ 611.9) could also be detected and the observed signal for **5_A_** increased in size. We therefore suspect that the reducing environment of this medium converts nitrate to nitrite in a metal-catalyzed reduction. To further probe this reduction, a sample containing [IrCl(COD)(IMes)] (20 mM), pyridine (3 equiv) and Na^15^NO_3_ (25 equiv) was exposed to 3 bar of H_2_ at 298 K for 24 h and the growth of the hydride ligand resonance for **5_A_** at δ_H_ −21.49 was monitored by thermally polarized ^1^H NMR spectroscopy over the course of 24 h. The resulting integral data for this peak could be fitted to an exponential growth curve (see the Supporting Information). After 24 h and refreshing the H_2_ atmosphere, further conversion to **5_A_** could again be seen which indicates that H_2_ is needed to drive this reaction. While the electrochemical reduction of nitrate is widely known^124,125^ and limited examples of heterogeneous hydrogenative reduction of nitrate are also reported,^126−128^ to the best of our knowledge the molecular reduction of nitrate using transition-metal catalysis has not received significant attention. Optimization of the phenomenon reported here may therefore provide a useful alternative.

## Conclusions

In this work, we have demonstrated how the ^15^N hyperpolarization of a range of important ^15^N-synthons, including some which feature in the important nitrogen cycle (NO_2_^–^ (28% polarization), NH_3_ (3%), PhCH_2_NH_2_ (5%), NaN_3_ (3%), and NO_3_^–^ (0.1%)), is possible. When monitored by ^15^N NMR, all these species yield strong signals that can be detected readily. The in-field, *T*_1_ values of NO_2_^–^ (17 s), ND_3_ (36 s), PhCH_2_NH_2_ (12 s), and NaN_3_ (50 s) mean that sufficient time exists to monitor their reactivity through hyperpolarized product responses. This has been demonstrated for the formation of phenyl diazonium, phenyl azide, a triazole, an amide, a sulfonamide, and two imines (Figure 4). In the case of phenyl azide formation, a pentazole intermediate was detected whose cyclic, rather than acyclic, formulation has been confirmed. Studies of the unlabeled formation of phenyl diazonium are also detailed.

Hence, these results demonstrate how SABRE reflects a versatile tool capable of tracking the preparation of a range of nitrogen rich products. We expect the future application of this approach to aid in achieving the optimized the synthesis of many materials, including important pharmaceuticals.

Furthermore, we demonstrate a biphasic method using a 15-crown-5 as a phase-transfer agent that yields >8% aqueous NO_2_^–^ polarization. We expect this route to help SABRE deliver biocompatible products in the future as we expect it to produce large amounts of such hyperpolarized reagents in seconds. The recent report of 79% ^15^N-derived SABRE hyperpolarization^62^ suggests that with further optimization a currently unrivaled low-cost approach to rapidly deliver ^15^N NMR sensitivity will therefore be obtained. Hence, the pioneering work of Bowers and Weitekamp again continues to expand beyond its original horizons.^129^

## Supporting Information Available

Full experimental methods, hyperpolarized NMR spectra, and characterization data (PDF)

## Abbreviations

SABRE: signal amplification by reversible exchange
*p*-H_2_: *para*-hydrogen
PHIP: parahydrogen induced polarization
d-DNP: dissolution dynamic nuclear polarization
PET: positron emission tomography
DMAP: dimethylamino pyridine
