# Supplementary material for: Real-Time High-Sensitivity Reaction Monitoring of Important Nitrogen-Cycle Synthons by 15N Hyperpolarized Nuclear Magnetic Resonance
Source: J Am Chem Soc. 2022 May 4;144(19):8756–69. doi: 10.1021/jacs.2c02619 (PMC9121385; doi:10.1021/jacs.2c02619)
Supplement: Supplementary file 1 — ja2c02619_si_001.pdf [file ja2c02619_si_001.pdf]

Supporting Information

Real time high sensitivity reaction monitoring of important nitrogen cycle synthons by ^{15}N hyperpolarized NMR

Peter J. Rayner[a], Marianna Fekete[a], Callum A. Gater[a], Fadi Ahwal[a], Norman Turner[b], Aneurin J. Kennerley[a], Simon B. Duckett[a*]

[a]Centre for Hyperpolarisation in Magnetic Resonance, Department of Chemistry, University of York, Heslington, York, YO10 5DD, United Kingdom

[b] University of Huddersfield, Queensgate, Huddersfield, West Yorkshire, HD1 3DH, United Kingdom

1 SABRE NMR Polarization transfer experiments

1.1 SABRE Polarization transfer method

The polarization transfer experiments that are reported were conducted in 5 mm NMR tubes that were equipped with a J. Young's tap. Samples for these polarization transfer experiments were based on a 5 mM solution of [IrCl(COD)(NHC)], co-ligand and the indicated additional substrate at the specified loading in methanol-d_4 or dichloromethane-d_2 (0.6 mL). The samples were degassed by two freeze-pump-thaw cycles prior to the introduction of *para*hydrogen at a pressure of 3 bar. *Para*-hydrogen (p-H_2) was produced by passing hydrogen gas over a spin-exchange catalyst (Fe_2O_3) at 28 K and used for all hyperpolarization experiments. This method produces constant p-H_2 with ca. 98% purity. Once filled with p-H_2, samples were shaken vigorously in the specified polarization transfer field before being rapidly transported into the magnet for subsequent interrogation by NMR spectroscopy.

1.2 Biphasic SABRE Polarization transfer method

Samples for polarization using biphasic[1] conditions were prepared as follows. [IrCl(COD)(NHC)] (5 mM), co-ligand, 15-crown-5 and substrate were dissolved in dichlormethane-d_2 (0.3 mL) in a 5 mm NMR tube that was equipped with a J. Young's tap. After degassing the sample using a freeze-pump-thaw method, the sample was exposed to 3 bar H_2 for 1 h prior to the subsequent addition of 0.3 mL of degassed D_2O inside a glove box filled with N_2. Once filled with p-H_2, samples were shaken vigorously in the specified polarization transfer field before being rapidly transported into the magnet for subsequent interrogation by NMR spectroscopy.

1.3 Calculation of Enhancement Factors

^1H signal enhancements were calculated according to equation 1 where, E = enhancement level, SI(pol) = signal of polarized sample, SI(unpol) = signal of unpolarized (reference) sample.

$$E = \frac{SI(pol)}{SI(unpol)} \qquad (1)$$

Experimentally, both spectra were recorded on the same sample using identical acquisition parameters, including the receiver gain. The raw integrals of the relevant resonances in the polarized and unpolarized spectra were then used to determine the enhancement levels. The quoted values reflect the signal strength gain (fold) per proton nucleus in the specified group. The reference sample was allowed to equilibrate within the NMR spectrometer for 1-2 minutes prior to acquisition.

Heteronuclear enhancement factors were determined by comparison to either spectra obtained of high concentration solutions or spectra obtained under signal averaging. Calculations were made using standard literature methods.[2]

1.4 Example NMR spectra

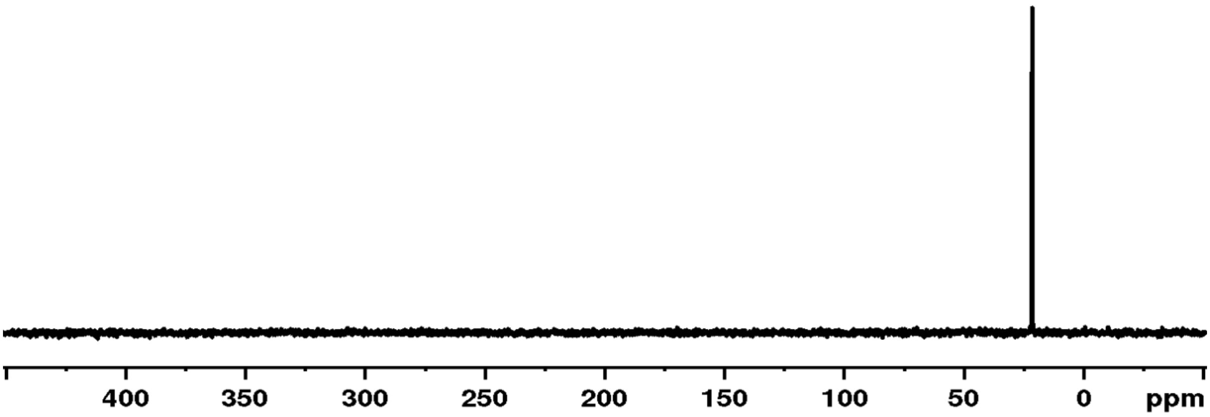

Figure S1 Reference: Single scan, thermally polarized ^{15}N NMR spectrum of a 5.0 M solution of $^{15}NH_4Cl$ in D_2O

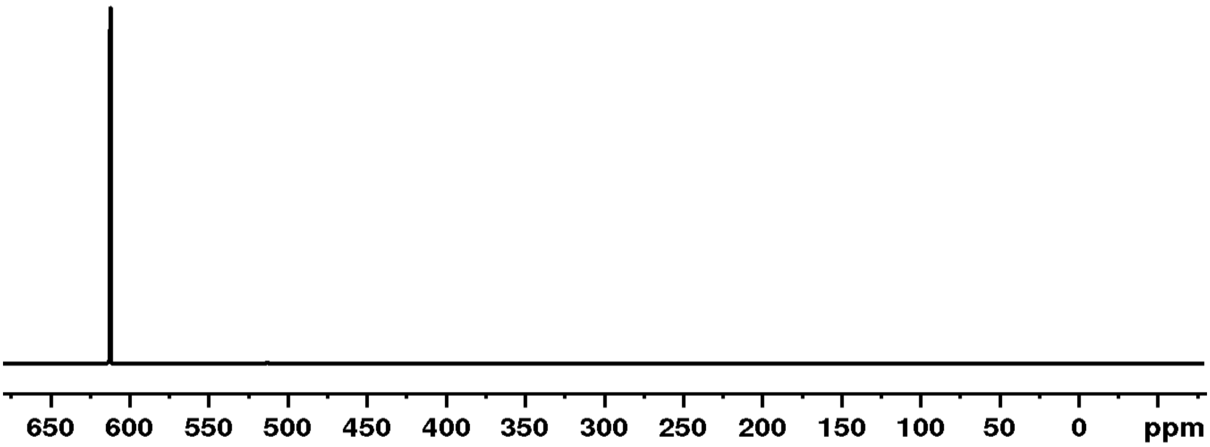

Figure S2 A single scan SABRE hyperpolarized ^{15}N NMR spectrum of a solution containing [IrCl(COD)(IPr^{NMe2})] (5 mM), $Na^{15}NO_2$ (25 eq.) and DMAP-d_2 (6 eq.) in methanol d_4 under 3 bar *p*-H_2 after polarization transfer at −3.5 mG.

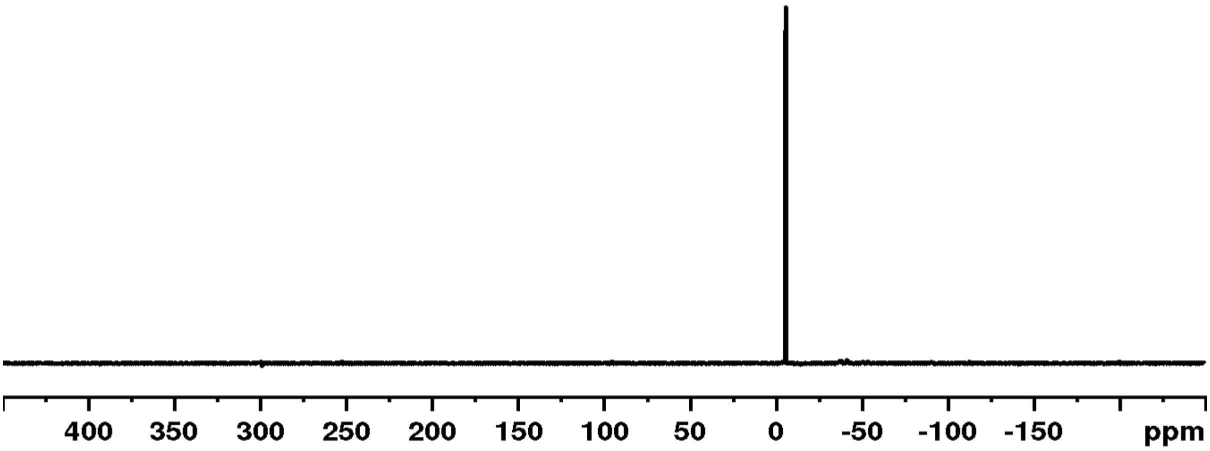

Figure S3 A single scan SABRE hyperpolarized ^{15}N NMR spectrum of a solution containing [IrCl(COD)(IMes)] (5 mM), $^{15}NH_4OH$ (10 eq.) and pyridine (3 eq.) in methanol d_4 under 3 bar *p*-H_2 after polarization transfer at −3.5 mG.

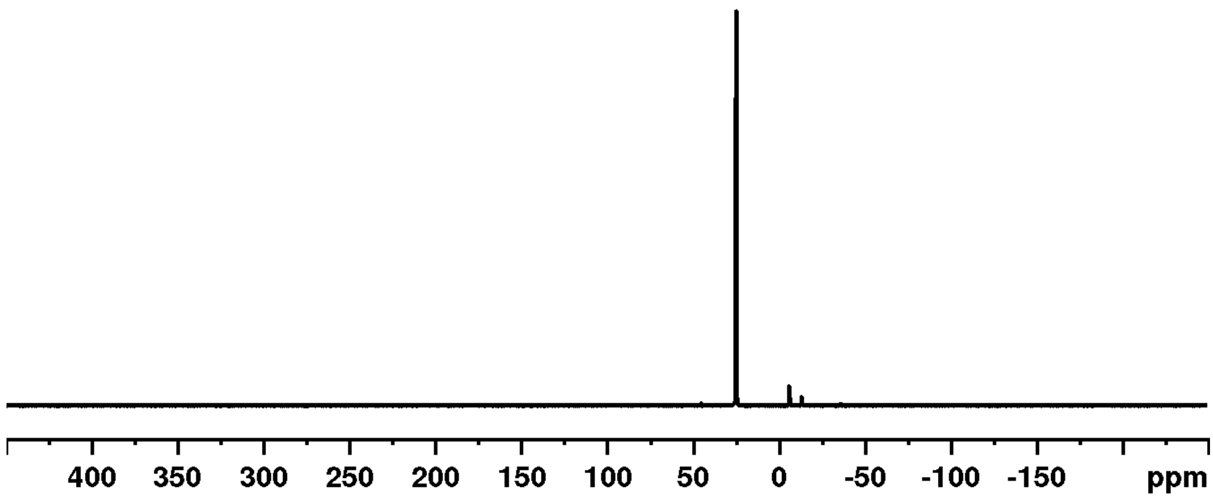

Figure S4 A single scan SABRE hyperpolarized ^{15}N NMR spectrum of a solution containing [IrCl(COD)(IMes)] (5 mM) and benzylamine-^{15}N (10 eq.) in dichloromethane-d_2 under 3 bar p-H_2 after polarization transfer at −3.5 mG.

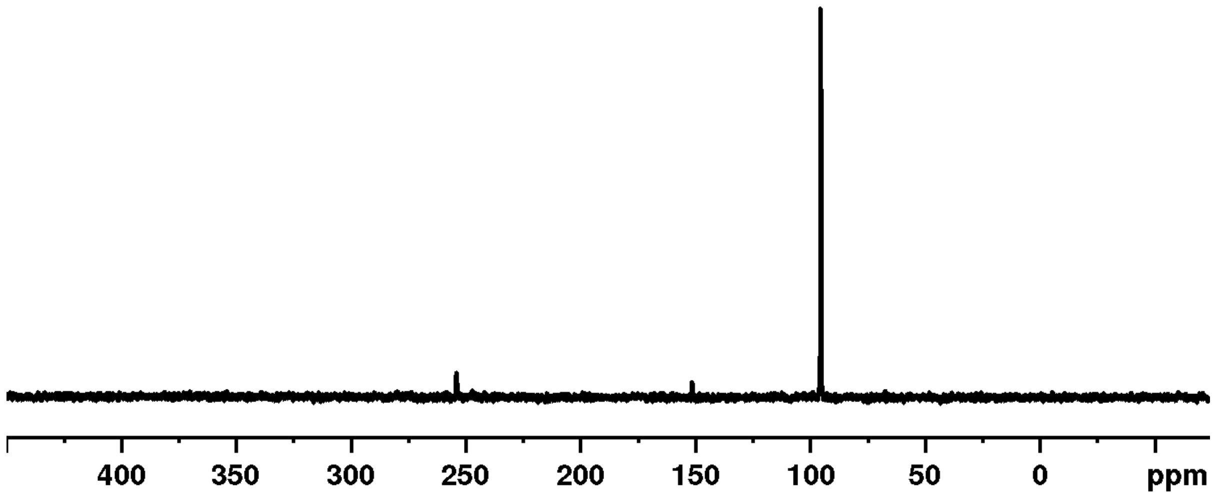

Figure S5 A single scan SABRE hyperpolarized ^{15}N NMR spectrum of a solution containing [IrCl(COD)(IMes)] (5 mM), 1-^{15}N-NaN_3 (10 eq.) DMAP (3 eq.) in methanol-d_4 under 3 bar p-H_2 and polarization transfer at −3.5 mG.

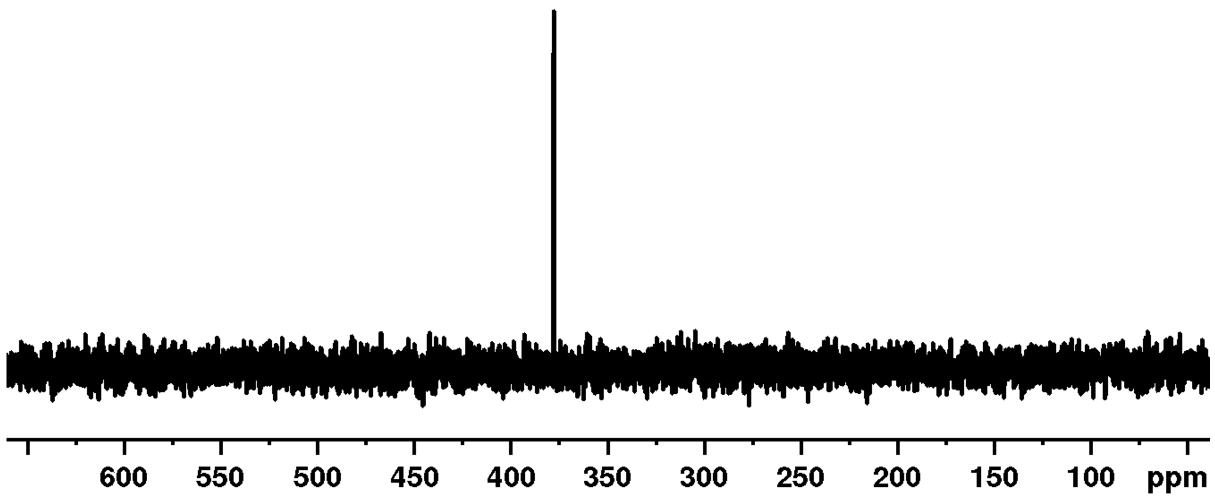

Figure S6 A single scan SABRE hyperpolarized ^{15}N NMR spectrum of a solution containing $[Ir(COD)(IMes)(pyridine)]BF_4$ (5 mM), $Na^{15}NO_3$ (10 eq.) DMSO-d_6 (3 eq.) in methanol-d_4 under 3 bar p-H_2 and polarization transfer at −3.5 mG.

Effect of Polarization Transfer Field

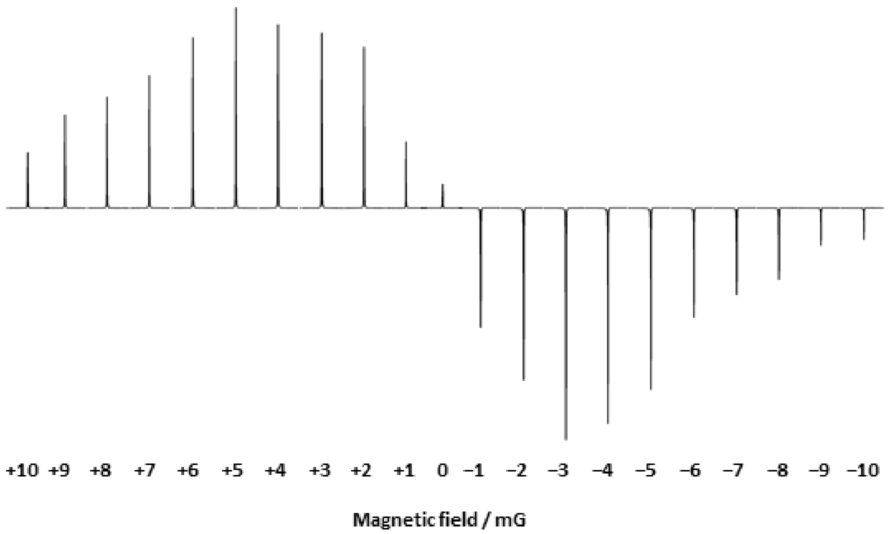

Figure S7: Effect of polarization transfer field on the SABRE derived polarization resulting for $Na^{15}NO_2$ in the presence of the co-ligand pyridine.

2 Effect of DMAP concentration on $Na^{15}NO_2$ signal enhancement

The effect of DMAP concentration on the ^{15}N NMR signal enhancement level of $Na^{15}NO_2$ was investigated. This involved using a series of 5 mm NMR tubes containing incrementally increasing equivalents of DMAP with fixed concentrations of [IrCl(COD)(IMes)] (5 mM) and $Na^{15}NO_2$ (125 mM) in methanol-d_4 (0.6 mL). Each sample was shaken in a −3.5 mG polarization transfer field for 20 seconds under 3 bar p-H_2 before being rapidly transferred into an NMR spectrometer for interrogation at 9.4 T. Figure S8 shows that at low concentrations of DMAP, the signal enhancements reduce. This is effect is likely to be caused by a reduction

in the concentration of DMAP meaning that exchange with the active species, $[Ir(H)_2(^{15}NO_2)(IMes)(DMAP)_2]$ slows. Increasing the number of equivalents of DMAP to 6 increased the signal enhancement. Very high loadings of DMAP act to reduce the observed signal gains, presumably, as they influence negatively the H_2 ligand exchange rate.

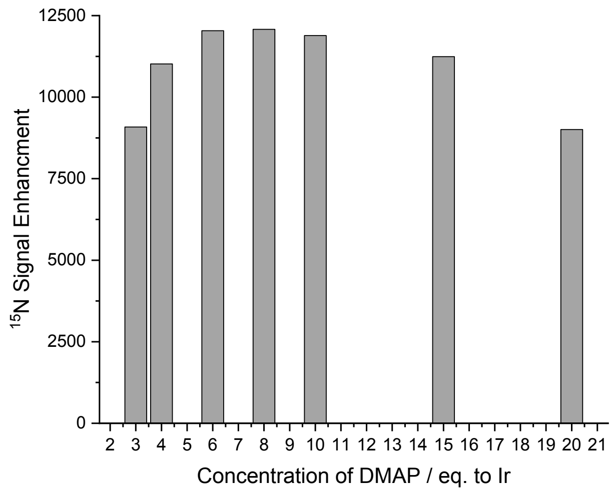

Figure S8: Effect of concentration of DMAP (expressed as equivalents) on the $Na^{15}NO_2$ signal enhancement after SABRE hyperpolarization at −3.5 mG using [IrCl(COD)(IMes)] (5 mM), DMAP (3-20 eq.) and $Na^{15}NO_2$ (25 eq.) in methanol-d_4 (0.6 mL).

3 Effect of $Na^{15}NO_2$ concentration of ^{15}N NMR signal enhancement

The effect of $Na^{15}NO_2$ concentration on the ^{15}N NMR signal enhancement of $Na^{15}NO_2$ was also investigated. A series of 5 mm NMR tubes containing incrementally increasing equivalents of $Na^{15}NO_2$ were prepared with a fixed concentration of [IrCl(COD)(IMes)] (5 mM) and DMAP (60 mM) in methanol-d_4 (0.6 mL). Each sample was shaken in a −3.5 mG polarization transfer field for 20 seconds under 3 bar p-H_2 before being rapidly transferred into an NMR spectrometer for interrogation at 9.4 T. Figure S9 shows that the highest signal gains are achieved for lower concentration of $Na^{15}NO_2$ and that increasing the number of equivalents of it relative to iridium reduces the signal enhancement.

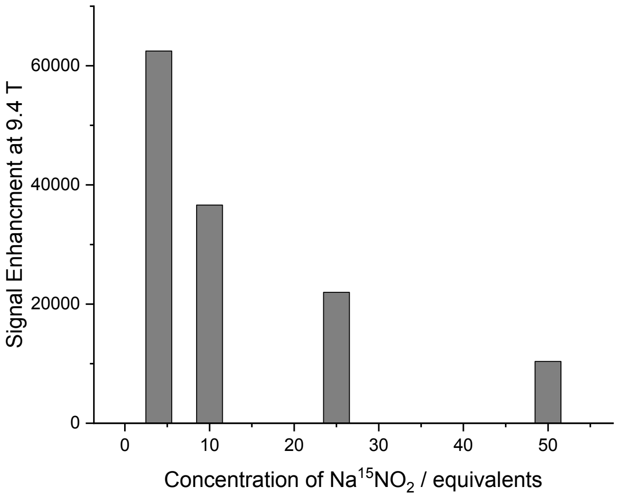

Figure S9: Effect of concentration of $Na^{15}NO_2$ on the ^{15}N NMR signal enhancement resulting after SABRE hyperpolarization at −3.5 mG using [IrCl(COD)(IMes)] (5 mM), DMAP (6 eq.) and $Na^{15}NO_2$ (4-50 eq.) in methanol-d_4 (0.6 mL).

4 Measurement of ligand loss rates using EXSY

The effect of the identity of the NHC ligand plays on the rate loss of the equatorially bound DMAP ligand from the active catalysts of type $[Ir(H)_2(NHC)(DMAP)_2(^{15}NO_2)]$ in methanol-d_4 was determined through the use of well-established EXSY methods. Integrals for the interchanging peaks in the associated ^1H EXSY spectra were obtained and converted into a percentage of the total detected signal. Table S1 summarises the data.

NHC	Rate of DMAP dissociation at 298 K / s^{-1}
IMes	0.128 ± 0.002
SIMes	0.425 ± 0.003
IPr	0.984 ± 0.004
IPent	3.421 ± 0.015
$IMes^{Cl}$	0.051 ± 0.001
$IMes^{Me}$	0.267 ± 0.008
$IMes^{NMe2}$	0.827 ± 0.009
IPr^{NMe2}	4.241 ± 0.012

Table S1: Effect of NHC ligand on the rate of DMAP dissociation from $[Ir(H)_2(NHC)(DMAP)_2(^{15}NO_2)]$

5 Optimization of ^{15}N SABRE polarization with $^{15}ND_3$

NHC	Signal Enhancement at 9.4 T
IMes	3765 ± 84
SIMes	3814 ± 254
IPr	3547 ± 97
IPent	2487 ± 58
IPr^{NMe2}	3687 ± 231

Table S2: Effect of NHC ligand on the ^{15}N NMR signal enhancement for$^{15}ND_3$ when using [IrCl(COD)(NHC)] (5 mM), $^{15}NH_4OH$ (10 eq.) in methanol-d_4 after polarization transfer in a −3.5 mG field.

Co-Ligand	Signal Enhancement at 9.4 T
Pyridine-d_5	15145 ± 154
DMSO-d_6	6816 ± 194
CD_3CN	5489 ± 97
$NaNO_2$	3251 ± 415
DMAP	7459 ± 255

Table S3: Effect of co-ligand on the ^{15}N NMR signal enhancement seen for $^{15}ND_3$ when using [IrCl(COD)(IMes)] (5 mM), $^{15}NH_4OH$ (10 eq.) and the specified co-ligand (5 eq.) in methanol-d_4 after polarization transfer at −3.5 mG field.

6 Preparation of hyperpolarized $Na^{15}NO_2$ in aqueous solution

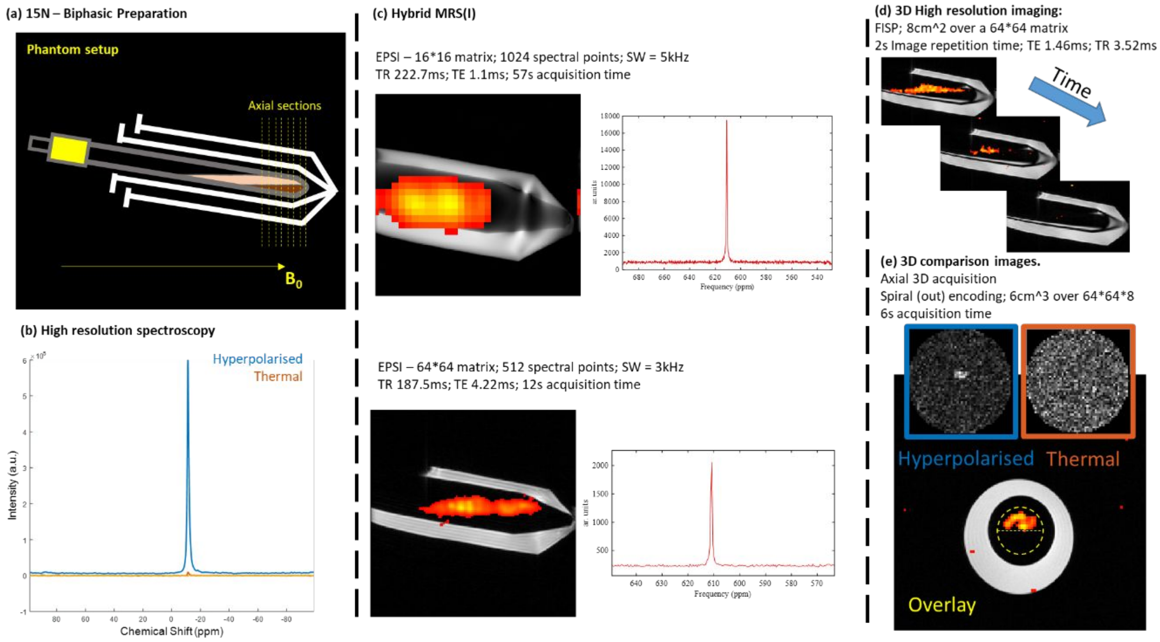

Figure S10 Demonstration of a ^{15}N-hyperpolarised aqueous bolus preparation under SABRE for NO_2^- (a) Picture of the sample used in the analysis; (b) demonstration that ^{15}N NO_2^- hyperpolarisation is achieved in the aqueous phase using phase transfer catalysis via a spatially resolved high-resolution 7 T spectrum; (c) two spatially resolved ^{15}N-images showing the response for the upper aqueous phase at different spatial resolutions; (d) time course map showing the decay of signal as a function of time after polarisation transfer; (e) thermally polarised comparison to demonstrate the need for a hyperpolarised response.

7 Reduction of Nitrate to Nitrite

An NMR tube containing a solution of [IrCl(COD)(IMes)] (5 mM), $Na^{15}NO_3$ (25 eq), pyridine (3 eq.) in methanol-d_4 was exposed to 3 bar H_2 and placed inside an NMR spectrometer at 298 K. Over the course of 24 h, the sample was periodically interrogated to produce a series of ^1H NMR spectra. The integral value of the hydride resonance for $[Ir(H)_2(^{15}NO_2)(IMes)(pyridine)_2]$ at δ_H−21.49 was monitored (Figure S10). These data show that reduction of nitrate to nitrite takes place under the reaction conditions. After 24 h, replenishing the H_2 atmosphere causes the reduction to continue.

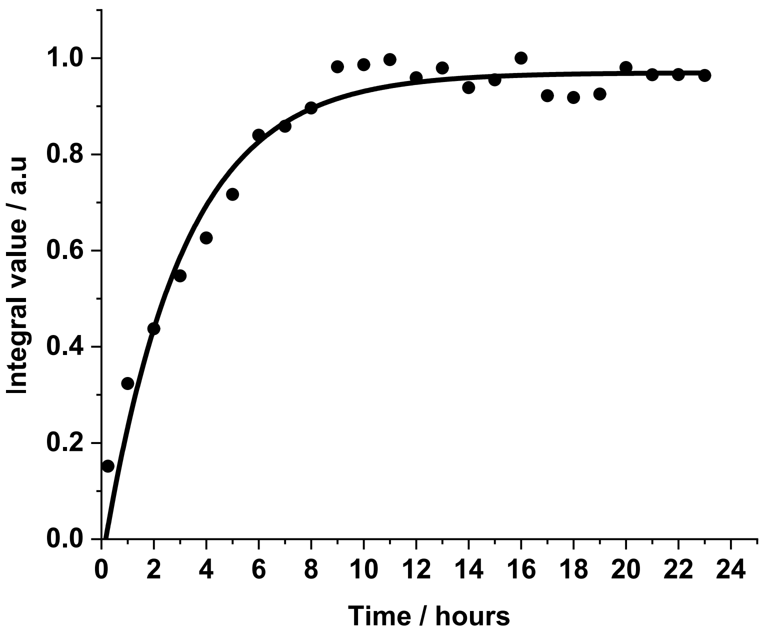

Figure S11 Growth of hydride resonance in the ^{1}H NMR spectrum at δ_H−21.49 over time after a sample containing [IrCl(COD)(IMes)] (5 mM), pyridine (6 eq.), $Na^{15}NO_3$ (25 eq.) in methanol-d_4 was exposed to 3 bar H_2.

8 Study of the creation of phenyl diazonium without ^{15}N labelling.

A sample was prepared that contained 5.6 mg of $NaNO_2$ in methanol-d_4 and hyperpolarized by SABRE as detailed in Figure S12 (a). The resulting ^{15}N signal was detected with a S/N ratio of 480. This sample was then exposed to unlabelled aniline in aqueous HCl and a single scan ^{15}N spectrum recorded ~15 seconds later. A signal for phenyl diazonium chloride was detected with S/N 24 as shown in Figure S12 (b). We conclude that when optimized, these methods could be employed for the analysis of unlabelled materials.

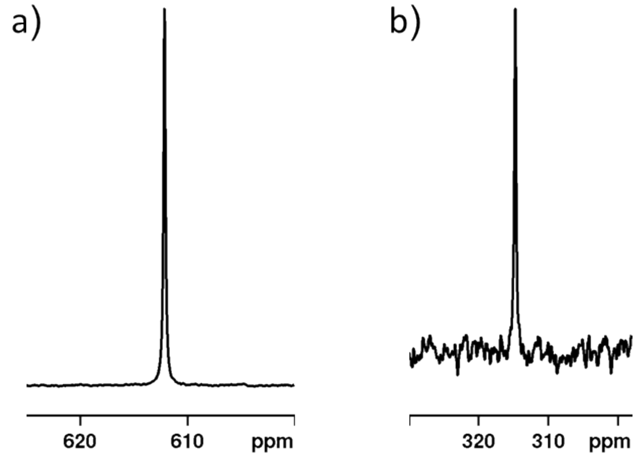

Figure S12: Hyperpolarized ^{15}N NMR spectra of the reaction between unlabelled $NaNO_2$ with aniline. a) Hyperpolarised ^{15}N NMR spectrum of unlabelled $NaNO_2$ hyperpolarized using [IrCl(COD)(IPr^{NMe2})] (5 mM), DMAP (6 eq.), $NaNO_2$ (25 eq.) in methanol-d_4. b) Resulting ^{15}N NMR spectrum of phenyldiazonium formed by reaction of hyperpolarized $NaNO_2$ and unlabelled aniline in HCl.

9 Synthesis and Characterisation of Novel Compounds

[IrCl(COD)(IMes^{NMe2})]

KO^{t}Bu (14 mg, 0.12 mmol, 2.4 eq.) was added to a stirred solution of IMes^{NMe2}.HOTf[3] (50 mg, 0.10 mmol, 2.0 eq) in THF (10 mL) at rt under N_2. The resulting suspension was stirred at rt for 30 min. Then, a solution of $[Ir(COD)Cl]_2$ (34 mg, 0.05 mmol, 1.0 eq.) was added and the resulting solution stirred at rt for 2 h. The solvent was removed under reduced pressure to give the crude product. Purification by flash column chromatography on silica with CH_2Cl_2 led to the isolation of [IrCl(COD)(IMes^{NMe2})] (61 mg, 89%) as a yellow crystalline solid, **^{1}H NMR** (400 MHz, $CDCl_3$) δ 7.03 (s,1H), 7.01 (s, 1H) 6.97 (s, 2H), 6.33 (s, 1H), 4.17 (td, J = 7.8 Hz, 3.9 Hz, 1H), 4.10-4.05 (m, 1H), 3.08-3.04 (m, 1H), 2.78 (td, J = 7.5 Hz, 3.2 Hz, 1H), 2.46 (s, 6H), 2.42 (s, 3H), 2.41 (s, 3H), 2.38 (s, 3H), 2.37 (s, 3H), 2.19 (s, 3H), 2.14 (s, 3H), 1.78-1.63 (m, 4H), 1.40-1.14 (m, 4H); **^{13}C NMR** (100.6 MHz, $CDCl_3$) δ 178.1 (s), 146.7 (s), 138.4 (s), 138.3 (s), 137.7 (s), 137.4 (s), 136.6 (s), 135.5 (s), 134.4 (s), 134.0 (s), 129.53 (s), 129.45 (s), 128.2 (s), 128.0 (s), 108.0 (s), 82.1 (s), 81.5 (s), 51.5 (s), 51.1 (s), 43.2 (s), 33.8 (s), 33.2 (s), 29.2 (s), 28.6 (s), 21.2 (s), 21.1 (s), 20.3 (s), 17.7 (s), 18.7 (s), 18.2 (s); **HRMS** m/z calculated for $C_{31}H_{41}{}^{193}IrN_3$ $(M - Cl)^+$ 648.2930, found 648.2947 (+2.6 ppm error).

[IrCl(COD)(IPr^{NMe2})]

KO^{t}Bu (135 mg, 1.2 mmol, 2.4 eq.) was added to a stirred solution of the IPr^{NMe2}.HOTf[3] (582 mg, 1.0 mmol, 2.0 eq) in THF (10 mL) at rt under N_2. The resulting suspension was stirred at rt for 30 min. Then, a solution of $[Ir(COD)Cl]_2$ (338 mg, 0.50 mmol, 1.0 eq.) was added and the resulting solution was stirred at rt for 2 h. The solvent was removed under reduced pressure to give the crude product. Purification by flash column chromatography on silica with CH_2Cl_2 led to the isolation of [IrCl(COD)(IPr^{NMe2})] (644 mg, 84%) as a yellow crystalline solid, **^{1}H NMR** (400 MHz, $CDCl_3$) δ 7.50 (t, J = 7.4 Hz, 1H), 7.45 (t, J = 7.5 Hz, 1H), 7.45-7.41 (m, 1H), 7.38-7.31 (m, 1H), 7.29-7.20 (m, 2H), 6.40 (s, 1H), 4.32-4.26 (m, 1H), 4.06-3.98 (m, 1H), 3.75-3.62 (m, 1H), 3.55-3.48 (m, 1H), 3.04-2.85 (m, 2H), 2.71-2.63 (m, 1H), 2.45 (s, 6H), 2.24-2.13 (m, 1H), 1.78-0.93 (m, 32H); **^{13}C NMR** (100.6 MHz, $CDCl_3$) δ 180.6 (s), 147.7 (s), 146.7 (s), 145.4 (s), 137.0 (s), 133.6 (s), 130.0 (s), 129.5 (s), 125.7 (s), 124.7 (s), 124.2 (s), 123.6 (s), 123.2 (s), 122.4 (s), 110.0 (s), 84.2 (s), 79.1 (s), 54.3 (s), 43.2 (s), 31.3 (s), 31.2 (s), 29.1 (s), 28.8 (s), 28.0 (s), 27.4 (s), 26.6 (s), 26.3 (s), 24.8 (s), 24.5 (s), 24.4 (s), 23.3 (s), 21.9 (s); **HRMS** m/z calculated for $C_{37}H_{53}{}^{193}IrN_3$ $(M - Cl)^+$ 732.3869, found 732.3894 (+3.4 ppm error).

^{15}N-benzyl-1,1,1-trifluoromethanesulfonamide

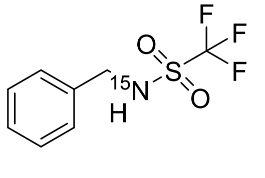

A solution of trifluoromethanesulfonyl chloride (32 µL, 0.19 mmol, 1.0 eq.) in CH_2Cl_2 (2 mL) was added dropwise to a solution of ^{15}N-benzylamine (25 µL, 0.23 mmol, 1.2 eq.) and Et_3N (1.2 eq.) in CH_2Cl_2 (5 mL) at 0 °C. The resulting solution was stirred at room temperature overnight. The solvent was removed under reduced pressure and the resulting residue was dissolved in EtOAc and washed with a 1 M solution of $HCl_{(aq)}$. The organic phase was dried ($MgSO_4$) and the solvent was removed under reduced pressure. ^{1}H NMR (CD_2Cl_2, 400 MHz): δ 7.44-7.36 (m, 5H), 5.74 (dt, J = 91.5, 5.8 Hz, 1H), 4.48 (d, J = 5.8 Hz, 2H); ^{13}C NMR

(CD_2Cl_2, 100 MHz): δ 135.6 (s, 1C), 129.0 (s, 2C), 128.5 (s, 1C), 127.8 (s, 2C), 119.8 (qd, J = 320.2, 9.0 Hz, 1C), 48.1 (d, J = 6.6 Hz, 1C); ^{15}N NMR (CD_2Cl_2, 43 MHz): δ 88.1 (d, 91.5 Hz); HRMS (ESI): m/z calculated for $C_8H_9F_3{}^{15}NO_2S$ 241.0271 $(M + H)^+$, found 241.0264 (error −2.9 ppm)

((5a*R*,6*R*,6a*S*)-1,4,5,5a,6,6a,7,8-Octahydrocyclopropa[5,6]cycloocta[1,2-d][1,2,3]triazol-6-yl)methanol

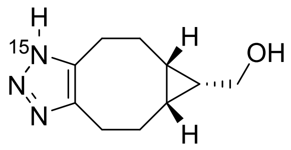

1-^{15}N-Sodium azide (3.6 mg, 0.055 mmol, 1.1 eq.) was added to a solution of (1R,8S,9s)-bicyclo[6.1.0]non-4-yn-9-ylmethanol (7.5 mg, 0.05 mmol, 1 eq.) in methanol-d_4 inside an NMR tube fitted with a J. Young's Tap. The NMR tube was placed in warm water for 30 min and then analysed by ^1H NMR spectroscopy. This showed completed conversion to the product had occurred. ^1H NMR (CD_3OH, 500 MHz): δ 3.67-3.64 (m, 4H), 3.09 (ddd, J = 14.5, 6.5, 2 Hz, 1H), 2.72 (ddd, J = 14.5,10.5, 2 Hz, 1H), 2.20-2.17 (m, 1H), 1.50-1.42 (m, 1H), 1.33-1.26 (m, 1H), 1.17-1.12 (m, 1H), 1.11-1.05 (m, 1H), 0.94-0.85 (m, 2H); ^{13}C NMR (CD_3OH, 125 MHz): δ 141.2, 58.5, 58.3, 28.6, 25.1, 23.3, 20.6, 20.5, 19.7; ^{15}N NMR (CD_3OH, 54 MHz): δ 321.1 HRMS (ESI): m/z calculated for $C_{10}H_{15}N_2{}^{15}NONa$ 217.1078 $(M + Na)^+$, found 217.1073 (error −2.1 ppm)

10 Characterisation data for the detected complexes

10.1 [Ir(COD)(IMes)(NO_2)] (2)

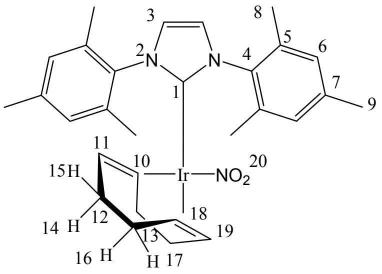

Resonance number	^1H (ppm)	$^{13}C\{^1H\}$ (ppm)	^{15}N (ppm)
**1**		174.2 (s, 1C)	
**2**			194.3
**3**	7.34 (s, 2H)	124.3 (s, 2C)	
**4**		155.0 (s, 2C)	
**5**		135.7 (s, 4C)	
**6**	7.09 (s, 4H)	128.6 (s, 4C)	
**7**		139.0 (s, 2C)	
**8**	2.22 (s, 12H)	17.4 (s, 4C)	
**9**	2.42 (s, 6H)	19.9 (s, 2C)	
**10/11**	3.79 (m, 2H)	119.6 (s, 2C)	
**12**		28.9 (s, 2C)	
**13**		32.1 (s, 2C)	
**14, 15**	1.90 (m, 2H), 1.52 (m, 2H)		
**16, 17**	1.80 (m, 2H), 1.58 (m, 2H)		
**18/19**	3.50 (m, 2H)	123.6 (s, 2C)	
**20**			490.7

10.2 [Ir(H)$_2$(η^2-COD)(IMes)(pyridine)($^{15}NO_2$)] (3)

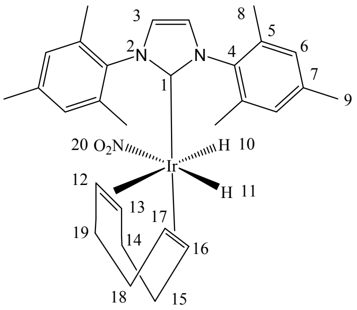

Resonance number	^{1}H (ppm)	^{13}C (ppm)	^{15}N (ppm)
**1**		154.78	
**2**			198.1
**3**	7.25 (s, 2H)	124.05	
**4**		119.13	
**5**		115.75, 115.61	
**6**	7.04 (br, 2H), 7.02 (br, 2H)	128.80, 128.50	
**7**		117.27	
**8**	2.07 (s, 6H), 2.30 (s, 6H)	16.74, 17.62	
**9**	2.36 (s, 6H)	19.20	
**10**	−14.17 (br s 1H)		
**11**	−18.77 (J_{NH} = 23.1 Hz, J_{HH} = 3.3 Hz, 1H)		
**12**	4.60 (m, 1H)	96.65	
**13**	4.28 (m, 1H)	92.74	
**14**	1.68 (m, 1H), 1.39 (m, 1H)	27.53	
**15**	2.02 (m, 1H), 1.74 (m, 1H)	27.03	
**16**	4.21 (m, 1H)	83.59	
**17**	4.95 (m, 1H)	77.54	
**18**	2.97 (m, 1H), 2.13 (m, 1H)	37.92	
**19**	2.32 (m, 1H), 2.10 (m, 1H)	29.69	
**20**			476.1

10.3 [Ir(H)$_2$(IMes)(pyridine)$_2$(^{15}NO$_2$)] (5$_A$)

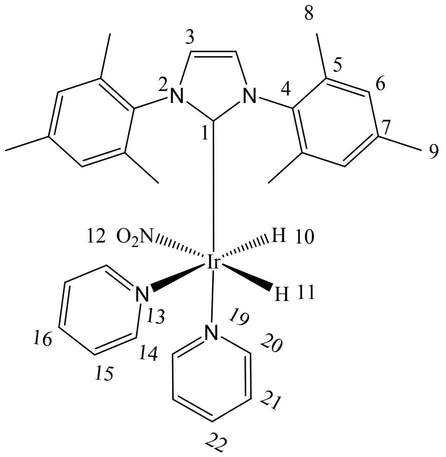

Resonance number	^{1}H (ppm)	^{13}C (ppm)	^{15}N (ppm)
**1**		121.89	
**2**			194.05
**3**	7.03 (s, 2H)	121.86	
**4**		138.01	
**5**		135.63, 135.58	
**6**	6.75 (s, 2H), 6.71 (s, 2H)	128.28	
**7**		137.85	
**8**	2.18 (s, 6H), 2.14 (s, 6H)	17.70, 17.13	
**9**	2.21 (s, 6H)	19.82	
**10**	−22.53 dd J_{NH} = 20.0 Hz, J_{HH} = -8 Hz		
**11**	−21.30 dd J_{NH} = 29.0 Hz, J_{HH} = -8 Hz		
**12**			511.1
**13**			258.3
**14**	8.62	154.13	
**15**	6.91	124.24	
**16**	7.60	134.38	
**19**			237.2
**20**	8.24	155.60	
**21**	6.94	124.40	
**22**	7.61	135.66	

10.4 Na[Ir(H)$_2$(IMes)(pyridine)($^{15}NO_2$)$_2$] (6_A)

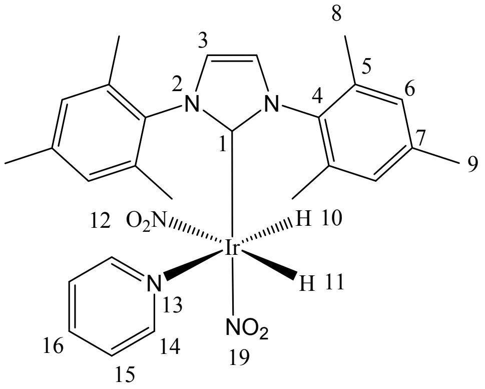

**Resonance number**	**^{1}H (ppm)**	**^{13}C (ppm)**	**^{15}N (ppm)**
**1**		122.17	
**2**			194.94
**3**	7.01 (s, 2H)	122.03	
**4**		137.72	
**5**		135.66, 135.51	
**6**	6.85 (s, 1H), 6.66 (s, 1H)	128.28, 128.25	
**7**		137.98	
**8**	2.11 (s, 6H), 2.08 (s, 6H)	17.05, 17.68	
**9**	2.26 (s, 6H)	19.86	
**10**	−22.05 (1H, dd J_{NH} = 20.0 Hz, J_{HH} = -7 Hz)		
**11**	−23.27 (1H dd J_{NH} = 29.0 Hz, J_{HH} = -7 Hz)		
**12**			509.2
**13**			254.5
**14**	8.76 (m, 2H)	154.87	
**15**	6.90 (m, 2H)	123.30	
**16**	7.595 (s, 1H)	134.06	
**19**			483.2

10.5 Na[Ir(H)$_2$(IMes)(DMSO)($^{15}NO_2$)$_2$] (6_G)

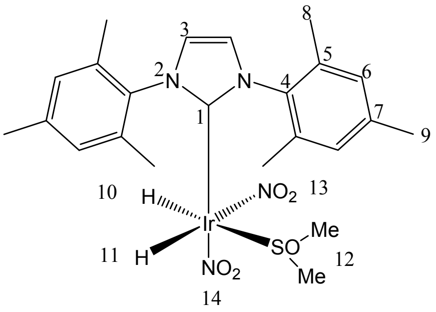

**Resonance number**	**^{1}H (ppm)**	**^{13}C (ppm)**	**^{15}N (ppm)**
1		118.0	
2			196.1
3	7.10 (s, 2H)	122.74	
4		135.5	
5		136.87, 137.22	
6	6.97 (s, 2H), 6.92 (s, 2H)	128.4, 128.6	
7		138.45	
8	2.17 (s, 6H), 2.22 (s, 6H)	18.26, 17.57	
9	2.36 (s, 6H)	19.96	
10	−22.17 (1H J_{NH} 29.6, J_{HH} 6.85)		
11	−15.57 (1H J_{HH} 6.85)		
12	2.98 (s, 3H), 2.92 (s, 3H)	40.14, 48.36	
13			500.65
14			468.72

10.6 Characterisation data for $Na[Ir(H)_2(IMes)(DMSO)(^{15}NO_2)_2]$ (7_G)

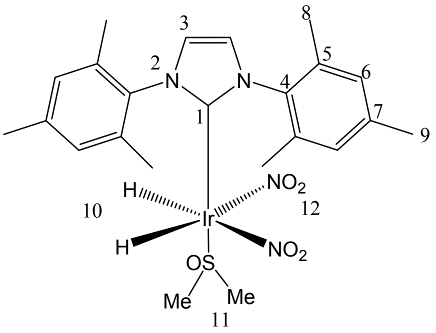

Resonance number	^1H (ppm)	^{13}C (ppm)	^{15}N (ppm)
1		117.0	
2			195.5
3	7.19 (s, 2H)	122.6	
4		135.86	
5		137.49	
6	6.87 (s, 4H)	128.2	
7		138.13	
8	2.15 (s, 12 H)	17.47	
9	2.315 (s, 3H)	20.0	
10	−22.32 (2H, JNHcis+JNHtrans = 27.6)		
11	3.16 (s, 6H)	49.70	
12			502.03

10.7 Partial characterization data for the minor product $[Ir(H)_2(IMes)(DMSO)_2(^{15}NO_2)]$ (5_G)

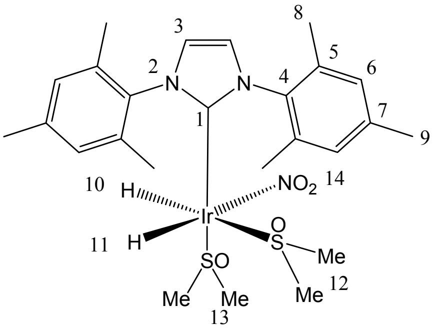

Resonance number	^1H (ppm)	^{13}C (ppm)	^{15}N (ppm)
3	7.30 (s, 2H)	123.67	
6	6.99 (s, 2H), 6.96 (s, 2H)	128.66	
8	2.19 (s, 6H), 2.17 (s, 6H)	18.11, 17.52	
9	2.41	19.92	
10	-16.08, (J_{HH} 6.0)		
11	-21.54 (J_{NH} 28.4, J_{HH} 6.0)		
12	3.18 (s, 3H), 2.80 (s, 3H)	42.21, 48.9	
13	3.42 (s, 3H), 3.06 (s, 3H)	57.56, 44.57	
14			486.3

10.8 Characterisation data for [Ir(H)$_2$(IPr^{NMe2})(pyridine)$_2$($^{15}NO_2$)]

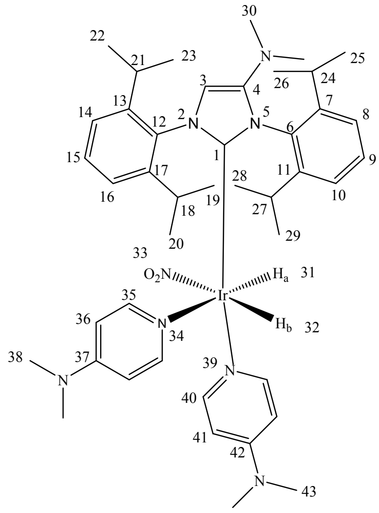

Resonance number	^{1}H (ppm)	^{13}C (ppm)	^{15}N (ppm)
**1**		153.25	
**2**			183.66
**3**	6.47	110.95	
**4**		144.14	
**5**			190.81
**6**		137.04	
**7**		146.71	
**8**		123.81	
**9**		128.36	
**10**		123.88	
**11**		146.45	
**12**		139.18	
**13**		145.98	
**14**		122.71	
**15**		128.57	
**16**		123.67	
**17**		145.99	
**18**	3.53	27.91	27.91
**19**	1.48	22.07	
**20**	1.23	25.61	
**21**	3.43	28.05	
**22**	1.24	25.61	
**23**	1.02	24.75	
**24**	3.25	28.54	
**25**	1.45	23.89	
**26**	1.19	23.52	
**27**	3.22	28.85	
**28**	1.46	23.44	
**29**	1.19	23.47	
**30**	2.92	17.73	
**31**	−22.27		
**32**	−21.16		
**33**			
**34**			217.2
**35**	7.81	153.62	
**36**	5.96	106.49	
**37**			
**38**	2.92	37.76	
**39**			196.1
**40**	7.69	154.88	
**41**	6.10	106.03	
**42**			
**43**	2.86	37.72	

11 References

1. Iali, W.; Olaru, A. M.; Green, G. G. R.; Duckett, S. B., Achieving High Levels of NMR-Hyperpolarization in Aqueous Media With Minimal Catalyst Contamination Using SABRE. *Chem. - Eur. J.* **2017,** *23*, 10491-10495.
2. Shchepin, R. V.; Jaigirdar, L.; Theis, T.; Warren, W. S.; Goodson, B. M.; Chekmenev, E. Y., Spin Relays Enable Efficient Long-Range Heteronuclear Signal Amplification by Reversible Exchange. *J. Phys. Chem. C* **2017,** *121* (51), 28425-28434.
3. Zhang, Y.; César, V.; Storch, G.; Lugan, N.; Lavigne, G., Skeleton Decoration of NHCs by Amino Groups and its Sequential Booster Effect on the Palladium-Catalyzed Buchwald–Hartwig Amination. *Angew. Chem. Int. Ed.* **2014,** *53* (25), 6482-6486.
